# Evolution of Matrix Gla and Bone Gla Protein Genes in Jawed Vertebrates

**DOI:** 10.3389/fgene.2021.620659

**Published:** 2021-03-10

**Authors:** Nicolas Leurs, Camille Martinand-Mari, Stéphanie Ventéo, Tatjana Haitina, Mélanie Debiais-Thibaud

**Affiliations:** ^1^ISEM, CNRS, IRD, EPHE, Univ. Montpellier, Montpellier, France; ^2^Institute for Neurosciences of Montpellier, Saint Eloi Hospital, Inserm UMR 1051, Univ. Montpellier, Montpellier, France; ^3^Department of Organismal Biology, Uppsala University, Uppsala, Sweden

**Keywords:** Gla protein, osteocalcin, shark, skeleton, evo-devo, biomineralization, bglap

## Abstract

Matrix Gla protein (Mgp) and bone Gla protein (Bgp) are vitamin-K dependent proteins that bind calcium in their γ-carboxylated versions in mammals. They are recognized as positive (Bgp) or negative (Mgp and Bgp) regulators of biomineralization in a number of tissues, including skeletal tissues of bony vertebrates. The Mgp/Bgp gene family is poorly known in cartilaginous fishes, which precludes the understanding of the evolution of the biomineralization toolkit at the emergence of jawed vertebrates. Here we took advantage of recently released genomic and transcriptomic data in cartilaginous fishes and described the genomic loci and gene expression patterns of the Mgp/Bgp gene family. We identified three genes, Mgp1, Mgp2, and Bgp, in cartilaginous fishes instead of the single previously reported Mgp gene. We describe their genomic loci, resulting in a dynamic evolutionary scenario for this gene family including several events of local (tandem) duplications, but also of translocation events, along jawed vertebrate evolution. We describe the expression patterns of *Mgp1*, *Mgp2*, and *Bgp* in embryonic stages covering organogenesis in the small-spotted catshark *Scyliorhinus canicula* and present a comparative analysis with Mgp/Bgp family members previously described in bony vertebrates, highlighting ancestral features such as early embryonic, soft tissues, and neuronal expressions, but also derived features of cartilaginous fishes such as expression in fin supporting fibers. Our results support an ancestral function of Mgp in skeletal mineralization and a later derived function of Bgp in skeletal development that may be related to the divergence of bony vertebrates.

## Introduction

Vertebrates display a range of skeletal tissues that are biomineralized through the regulation of calcium phosphate crystal deposition (Janvier, 1996; Donoghue and Sansom, 2002; Omelon et al., 2009), except in the extant cyclostome group (agnathan fishes: lampreys and hagfishes) where the skeletal units are made of cartilage with no detection of calcium precipitates (Yao et al., 2011; Ota et al., 2013). Several vitamin K-dependent (VKD) proteins were shown to be involved in skeletal tissue mineralization in jawed vertebrates (reviewed in Bordoloi et al., 2018; Wen et al., 2018). Of these, Mgp (matrix Gla protein) and Bgp (bone Gla protein, bglap, and osteocalcin) display consistent similarities in their sequences and were considered to belong to the same gene family (Laizé et al., 2005; Cancela et al., 2014). Both these proteins display a Gla domain characterized by the ability to undergo γ-carboxylation of several glutamate residues, resulting in a putative ability of the protein to bind calcium (reviewed in Yáñez et al., 2012).

Expression of the *Mgp* and *Bgp* genes in mouse first appeared spatially exclusive, with *Bgp* expressed uniquely in osteoblasts or osteocytes but also in odontoblasts, while *Mgp* expression was restricted to hypertrophic chondrocytes (Ikeda et al., 1992; D’Errico et al., 1997). More recent data support the expression of *Mgp* in other skeletal cells, including osteoblasts and osteoclasts (Coen et al., 2009). *Mgp* was also shown to be largely expressed in many soft tissues such as kidney, lung, heart, and spleen (Fraser and Price, 1988). Mgp was shown to act as an inhibitor of several processes both in skeletal and soft tissues: calcium precipitation in hyaline cartilage and human vascular smooth muscle cells (Luo et al., 1997; Schurgers et al., 2007; reviewed by Wen et al., 2018), and also dentin or bone matrices mineralization and osteoclast differentiation (Kaipatur et al., 2008; Zhang et al., 2019). The Bgp protein, on the other hand, seems to function in two ways, either in its carboxylated form by regulating hydroxyapatite crystal growth in skeletal tissues or in its non-carboxylated form by potentially acting as a circulating hormone that may be involved in energy metabolism and other functions (Diegel et al., 2020). Focusing on their skeletal functions, the data gathered from mammals indicate that Bgp is involved in the regulation, both positive and negative, of biomineralization processes in bone tissues, while Mgp is an inhibitory protein for these biomineralization processes (reviewed in Wen et al., 2018).

The evolutionary history of the Mgp/Bgp gene family has been discussed for more than two decades, particularly in relation to the evolution of a mineralized skeleton in vertebrates (Rice et al., 1994; Cancela et al., 2001, 2014; Pinto et al., 2001; Simes et al., 2003; Laizé et al., 2005; Gavaia et al., 2006; Viegas et al., 2013). The search for *Mgp* and *Bgp* genes in a variety of bony vertebrates led to the identification of two *Bgp* copies in several teleost fishes [the most recently identified being named *OC2* (Laizé et al., 2005; Cancela et al., 2014; Cavaco et al., 2014)] and also in some tetrapods [amphibians and sauropsids, where the recently identified duplicate was named *OC3* (Cancela et al., 2014)] while *Mgp* was, until now, only found to be present as a single gene (Cancela et al., 2014). The hypothesis was raised that *Mgp* and *Bgp* genes originated from an ancestral gene after the two whole-genome duplications in vertebrates (Laizé et al., 2005) and that more recent events of duplication of *Bgp* occurred more recently and independently in the bony fish and the tetrapod lineages (Cancela et al., 2014). Cartilaginous fishes, e.g., sharks (selachians), skates and rays (batoids), and holocephalans, are crucial in this issue as their lineage diverged from bony fishes more than 450 million years ago and they display a skeleton devoid of bone tissue but made of hyaline and mineralized cartilage (Janvier, 1996). Several authors have previously described the presence of a *Mgp* gene in two shark species, the school shark *Galeorhinus galeus* (Rice et al., 1994) and the blue shark *Prionace glauca* (Ortiz-Delgado et al., 2006) for which they showed high conservation with tetrapod for (i) the Mgp amino-acid motifs which are critical for post-translational modifications [serine phosphorylation and glutamate γ-carboxylation (Price et al., 1994; Ortiz-Delgado et al., 2006)]; (ii) *Mgp* expression pattern and Mgp sites of accumulation [vertebral cartilage, endothelium, kidney, heart (vascular endothelia and smooth muscle), and dentinal matrix (Ortiz-Delgado et al., 2006)]. Previous studies have not identified any sequence that would be homologous to *Bgp* in cartilaginous fish genomes (Cancela et al., 2014).

The current explosion of genomic data, including in the cartilaginous fish lineage, allows the better description of gene complement and gene expression in this *Mgp*/*Bgp* family. Here we collect transcriptomic and genomic data from different jawed vertebrates, including several cartilaginous fishes where we identify an unknown diversity of *Mgp*/*Bgp* sequences and their genomic loci. We describe their gene expression patterns in embryonic stages of the small-spotted catshark *Scyliorhinus canicula* and uncover highly conserved but also previously unknown sites of expression.

## Materials and Methods

### Collection of *Mgp/Bgp* Sequences in the Genomes and Transcriptomes of Chondrichthyans

#### Synteny Analyses

Matrix Gla protein and bone Gla protein sequences for human, mouse, and zebrafish were collected from GenBank and were used to screen locally assembled small-spotted catshark (*Scyliorhinus canicula*) and thornback ray (*Raja clavata*) transcriptomic data (Debiais-Thibaud et al., 2019) as well as the most recently assembled genome for *S. canicula* (sScyCan1.1, GCA_902713615.1), using TBLASTN. Additional cDNA sequences were obtained by screening accessible transcriptomic data collected by the SkateBase project^1^ [little skate *Leucoraja erinacea* transcriptome (Contig Build-2, GEO:GSM643957) and small-spotted catshark transcriptome (GEO:GSM643958)] using TBLASTN. Small-spotted catshark, little skate, and thornback ray sequences were then used to screen other databases for elephant shark genome assembly (GCA_000165045.2 Callorhinchus_milii-6.1.3) (Venkatesh et al., 2014) and whale shark genome *Rhincodon typus* (GCA_001642345.2 ASM164234v2) (Tan et al., 2019). Thornback ray, small-spotted catshark, and little skate cDNA sequences were used to map synteny on the thorny skate *Amblyraja radiata* and the smalltooth sawfish *Pristis pectinata* draft assembled genomes using TBLASTN [data accessed from, and analyzed in agreement with, Vertebrate Genome Project (Rhie et al., 2020), PriPec2.pri, GCA_009764475.1]. Syntenic genes in chondrichthyans are *Ddx47* (elephant shark XM_007909802.1 in GenBank; thorny skate ENSARAT00005031107 in Ensembl Rapid Release) and *Erp27* (elephant shark XM_007909813.1 in GenBank; thorny skate ENSARAT00005031079 in Ensembl Rapid Release). Synteny data in bony fish genomes were extracted from the Ensembl database for selected genomes [human genome assembly: (GRCh38.p10); mouse *Mus musculus* (GRCm38.p6); chicken *Gallus gallus* (GRCg6a); tropical clawed frog *Xenopus tropicalis* (Xenopus_tropicalis_v9.1); elephant shark *Callorhinchus milii* (Callorhinchus_milii-6.1.3); gar *Lepisosteus oculatus* (LepOcu1); zebrafish *Danio rerio* (GRCz10); Chinese softshell turtle *Pelodiscus sinensis* (PelSin_1.0); central bearded dragon *Pogona vitticeps* (pvi1.1); reedfish *Erpetoichthys calabaricus* (fErpCal1.1); Asian bonytongue *Scleropages formosus* (fSclFor1.1)] and from NCBI for the caecilian *Microcaecilia unicolor* (aMicUni1.1).

#### Phylogenetic Reconstruction

Protein sequences for all identified Mgp and Bgp genes from the different chondrichthyan species together with sequences from osteichthyan species were used for phylogenetic tree reconstruction. These protein sequences are preproteins as they are obtained from the translation of either the cDNA sequence or of a predicted gene from available genomes. All sequences used in this study are detailed with IDs and origin in the Supplementary Material 1. Sequences were aligned using MAFFT (Katoh et al., 2002; Katoh and Standley, 2013) using standard parameters (Supplementary Material 2). Because a large proportion of the sequences is predicted from genomes and may include false exons, this alignment was then cleaned using HmmCleaner with standard parameters (option “–large”) to remove low similarity segments (Di Franco et al., 2019). Our final alignment used for subsequent phylogenetic reconstruction was 129 amino-acid long and is available in the Supplementary Material 3. Phylogenetic analyses were performed on the amino-acid alignment to infer the evolutionary history of these genes. This data set was used to reconstruct gene phylogenies in Maximum Likelihood using IQ-TREE 1.6.1 (Nguyen et al., 2015) under the JTT + I + G4 evolution model for amino-acid data. Node support was estimated by performing a thousand ultra-fast (UF) bootstrap replicates (Hoang et al., 2017) and single branch tests (SH-aLRT; Guindon et al., 2010).

#### Protein Domain Description

Conservation of protein domains was evaluated by mapping previously identified functional regions (Laizé et al., 2005) onto the aligned sequences of human, mouse, chicken, zebrafish, elephant shark, and small-spotted catshark Mgp or Bgp proteins. Additional motif recognition was validated on the small-spotted catshark and elephant shark protein sequences with InterPro (Finn et al., 2017), SMART (Letunic et al., 2021), and FIMO version 5.3.0 (Grant et al., 2011).

#### Reconciliation Between the Gene Phylogeny and Species Phylogeny

Evolutionary scenario for gene duplication/loss was built minimizing the duplication and loss score with standard parameters in Treerecs (Comte et al., 2020), using contracted versions of the gene and species trees (Supplementary Material 4).

### *In situ* Hybridization and Histology

Identified small-spotted catshark *Mgp1*, *Mgp2*, and *Bgp* cDNA sequences were used to design the following primers (sequences are given in the 5′-3′ orientation): Fw TCACAGATTCACACTCGCTG and Rv GGCCGAACCAGAGC TGCTG amplifying 702 bp for *Mgp1*; Fw CCGATCTCAC AAACTGAGCT and Rv CACAGACTGCAGCAAATAGT amplifying 817 bp for *Mgp2*; Fw CCAGAGAAGATGATGG TCCT and Rv GGGGAATTAACAGAGTCGTC amplifying 675 bp for *Bgp.* Sequences were amplified from cDNA reverse-transcribed from total RNA extractions of a mix of embryonic stages. These PCR products were ligated into the pGEM-T easy vector using the TA cloning kit (Promega). Inserts with flanking T7 and SP6 sites were amplified using M13F/M13R primers and sequenced to verify the amplicon sequence and orientation, and these PCR products were then used as templates for the synthesis of antisense DIG riboprobes [3 μl reaction, 100–200 ng PCR product, DIG RNA labeling mix (Roche) with either T7 or SP6 (depending on the amplicon orientation) RNA polymerase (Promega), following manufacturer’s instructions]. Before *in situ* hybridization, all DIG-labeled riboprobes were purified on MicroSpin G50 column (GE Healthcare). The obtained expression patterns were different for each probe, excluding detectable cross-hybridization between Mgp1/Mgp2/Bgp probes, so we did not use sense probes as negative control.

Whole embryos of either 6 cm total length, 7.7 cm total length, or 9 cm long hatchlings, were euthanized in buffered tricaïne, eviscerated and fixed for 48 h in 4% paraformaldehyde in 1× phosphate-buffered saline (PBS) solution at 4 °C, rinsed in PBS 1× for an hour, and then transferred in 50% ethanol (EtOH)-PBS 1×, 75% EtOH-PBS 1×, and three successive bathes of 100% EtOH before storage at −20°C in EtOH 100%. We then sampled (i) the lower jaw (hatchling) and (ii) transversal slices in the posterior zone of the branchial arches to allow visualization of gene expression in, respectively, (i) teeth and the Meckel’s cartilage; and (ii) abdominal vertebrae, pectoral fin, or branchial rays. Experiments of *in situ* hybridization were performed on 14 μm thick cryosections of the chosen samples that had been progressively transferred back to PBS 1×, then equilibrated in sucrose 30% for 24 h before being transferred and frozen in Tissue-Tek^®^ O.C.T.^TM^ (Sakura Finetek France SAS). Consecutive cryosections were distributed on 10 successive slides to a maximum of 6–8 sections per slide and were stored at −20°C. *In situ* hybridization on sections was performed as described previously (Enault et al., 2015) with stringent conditions of hybridization at 70°C. *In situ* hybridization results were taken with Hamamatsu NanoZoomer 2.0-HT Slide Scanner (Montpellier RIO Imaging facility, INM Optique) with a 40× objective.

Histological staining (Hematoxylin-Eosine-Saffran) was performed at the local histology platform (RHEM platform at IRCM, Montpellier) on 7 μm paraffin sections of non-demineralized samples on a histology automaton.

### RNA Isolation and qPCR Analysis

Early embryos (three or four for each stage) were collected from embryonic stages 18–32 (Ballard et al., 1993), with stage 32 embryos <3.5 cm total length. Total RNA was isolated with ReliaPrep RNA tissue Miniprep system according to the supplier’s instructions (Promega), and their quality was verified on a Bioanalyzer 2100 instrument (Agilent): 500 ng of total RNA were used for cDNA preparation performed by Superscript II reverse transcriptase (Invitrogen) with an oligodT primer.

For quantitative PCR, 1:20 dilution of each cDNA was run in triplicate on a 384-well plate for each primer pair by using thermal cycling parameters: 95°C for 2 min, 95°C for 10 s, 68°C for 10 s, 72°C for 10 s (45 cycles), and an additional step 72°C for 10 min performed on a Light Cycler 480 with the SensiFAST SYBR No-ROX kit (Meridian Bioscience) (qPHD UM2/GenomiX Platform, Montpellier – France). Results were normalized with the expression of two reference genes *Eef1a* and *Rpl8* [previously used in elasmobranch fishes (O’Shaughnessy et al., 2015; Onimaru et al., 2016)] by geometric mean, and data were further analyzed with the Light Cycler 480 software 1.5.1.

We used Primer 3.0 to design all the sets of forward and reverse primers to amplify selected genes (sequences are given in the 5′-3′ orientation): Fw TCGGGAGGAGAGATGCACAT and Rv TGCCACCAAAGTATCTGCCA amplifying 183 bp for *Mgp1*; Fw CCTGATTCTGCTGTGCCTGT and Rv TTTTCCATAGGC CGCCATGT amplifying 277 bp for *Mgp2*; Fw TGATGGT CCTTTCCTCGGGA and Rv TGGTATCCAATCCTGTTTGC CA amplifying 180 bp for *Bgp*; Fw GGTGTGGGTGAATTT GAAGC and Rv TTGTCACCATGCCAACCAGA amplifying 245 bp for *Eef1a*; Fw TTCATTGCAGCGGAGGGAAT and Rv TCAATACGACCACCACCAGC amplifying 302 bp for *Rpl8.*

The expression data obtained were compared over time to test if any gene was differentially expressed in time with a one-way ANOVA. A Shapiro–Wilk normality test was applied on the log transformed data, and for each gene the null hypothesis of normality was kept (*P* > 0.05). We tested for heteroscedasticity of variance between developmental stages, and the null hypothesis had to be rejected only for the Bgp gene (*P* < 0.05), even after log transformation. Note that we are very constrained by an unbalanced protocol (different number of observations in each developmental stage) and small sample size, which limits statistical power.

### Embryo Collection and Ethics Statement

Embryos of the small-spotted catshark *S. canicula* originated from a Mediterranean population of adult females housed at Station Méditerranéenne de l’Environnement Littoral, Sète, France. Handling of small-spotted catshark embryos followed all institutional, national, and international guidelines [European Communities Council Directive of September 22, 2010 (2010/63/UE)]: no further approval by an ethics committee was necessary as the biological material is embryonic and no live experimental procedures were carried out. Embryos were raised in seawater tanks at 16–18 °C and euthanized by overdose of tricaine (MS222, Sigma) at appropriate stages (Ballard et al., 1993; Enault et al., 2016).

## Results

### Evolution of the Mgp/Bgp Gene Complement in Jawed Vertebrates

**FIGURE 1:**
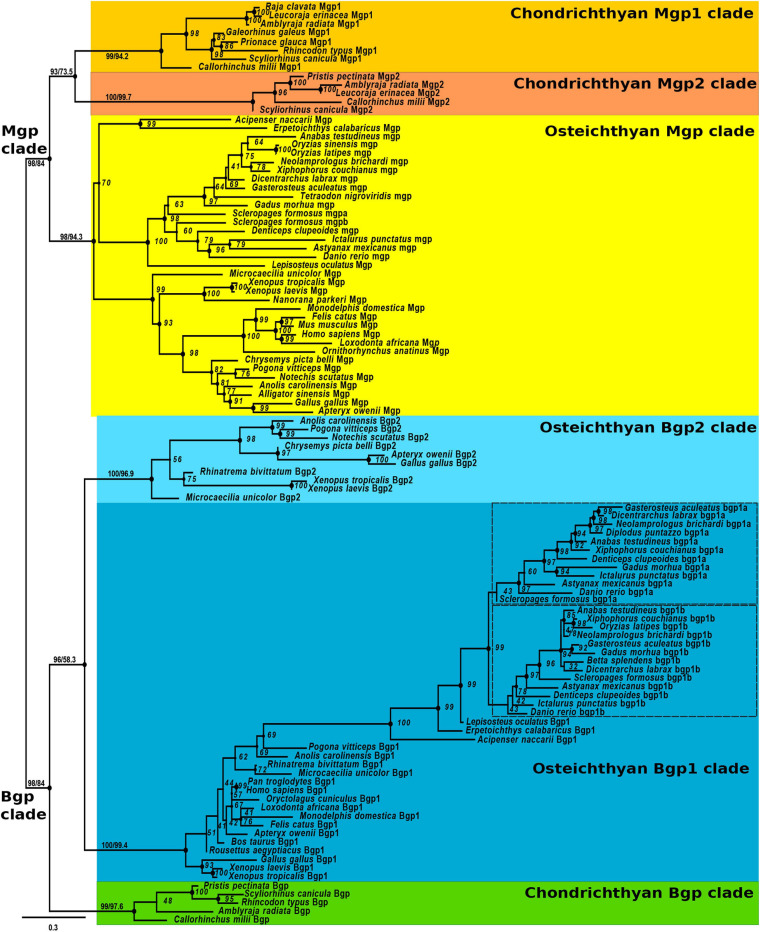
Maximum likelihood phylogenetic tree based on Bgp and Mgp amino-acid sequences (107 sequences, 129 positions) with JTT + I + G4 evolution model in IQ-TREE. Node support was evaluated with 1000 ultra-fast bootstrap replicates (shown on all nodes) and SH-aLRT (UFbootstrap/SH-aLRT), shown only on deeper nodes. Colored boxes indicate osteichthyan and chondrichthyan monophyletic clades. See text for gene name nomenclature.

Three transcripts were identified as Mgp or Bgp genes in the small-spotted catshark transcriptome and named after their position in the phylogenetic reconstruction: *Mgp1*, *Mgp2*, and *Bgp* (Figure 1). To perform this reconstruction, we screened other available cartilaginous fish genomes as well as the genomes of several bony fishes by reciprocal blasts to recover a maximum of Mgp/Bgp sequences in the jawed vertebrate clade. The produced alignment was 129 amino acid long after HmmCleaner (alignment available as Supplementary Material 3). The major limitation on the analysis of this phylogeny was the lack of an out-group: no potential Mgp/Bgp sequence could be identified in the available genomic and transcriptomic sequences for cyclostome species (e.g., lamprey or hagfish), and there is currently no identified closely related gene family in jawed vertebrates. Both Mgp and Bgp clades in bony fishes were monophyletic and had a closest monophyletic group made of cartilaginous fish sequences (see Supplementary Material 5 for the unrooted tree), which made us place the putative root of this tree as resulting in Figure 1, leading to one Mgp and one Bgp clade for jawed vertebrates. This choice implies that one ancestral Bgp and one ancestral Mgp genes were already present in the last common ancestor of extant jawed vertebrates, as previously suggested (Laizé et al., 2005).

In this phylogenetic reconstruction, two cartilaginous fish genes were identified as duplicated copies grouping together as the sister group to a single *Mgp* copy in bony fishes (UF-bootstrap and SH-aLRT support reach acceptable values at this chondrichthyan node, although they are lower than for other deep nodes): the two chondrichthyan copies were named *Mgp1* and *Mgp2* (Figure 1). In the Bgp clade, cartilaginous fish sequences were monophyletic and strongly supported by the SH-aLRT statistic and UF-bootstrap, with only one Bgp gene in each species, whereas bony fish sequences grouped into two sister clades, suggesting two osteichthyans Bgp paralogs well supported by the UF-bootstraps and SH-aLRT (Figure 1). One of these bony fish paralogs is best known as the *osteocalcin*/*Bgp* gene product in all screened actinopterygians and sarcopterygians (also previously named *OC1*; Cancela et al., 2014). To account for the different nature of the described paralogs, we will further identify this clade as Bgp1: although our phylogenetic reconstruction leads to little resolution within this clade, its monophyly is very robust in the tree (SH-aLRT = 99.4; UFboot = 100). The second osteichthyan Bgp paralog is herein named Bgp2: it includes sequences found only in lissamphibians and sauropsids (including birds). This *Bgp2* gene is predicted but most frequently not annotated in the Ensembl or NCBI databases (for *Chrysemys*, the kiwi bird and the tiger snake) or named *Mgp-like* in *Pogona, osteocalcin-like* in *Xenopus laevis* and other lissamphibians, *osteocalcin* in *X. tropicalis* or *osteocalcin 3* in the chicken [see all references to the extracted sequences in Supplementary Material 1; this paralog has also previously been named *OC3* (Cancela et al., 2014)]. As a consequence, this topology suggests an event of duplication of an ancestral bony fish *Bgp* gene leading to these *Bgp1* and *Bgp2* paralogs (Figure 1). Another event of duplication is deduced from the two sister clades observed within teleost fishes in the Bgp1 group: this and synteny data (see below) support these paralogs to originate from the teleost-specific whole-genome duplication (Amores et al., 1998), so we followed the accepted gene nomenclature and named them bgp1a (usually annotated *bglap* or *osteocalcin* in public databases) and bgp1b [previously named *OC2* (Cancela et al., 2014), or *bglap-like* in databases, see Supplementary Material 1].

### Genomic Organization of the Mgp and Bgp Genes in Jawed Vertebrates

**FIGURE 2:**
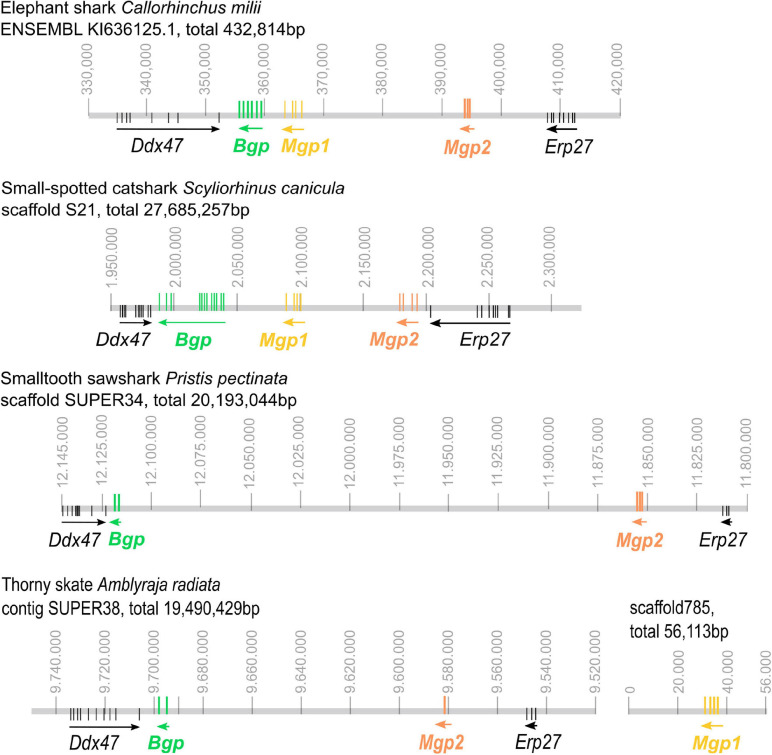
Genomic organization of the Mgp/Bgp gene clusters in reference chondrichthyan genomes: the elephant shark *Callorhinchus milii*; the small-spotted catshark *Scyliorhinus canicula*; the smalltooth sawfish *Pristis pectinata*; the thorny skate *Amblyraja radiata*. *Ddx47* and *Erp27* were included to insure the identification of homologous regions of the genome. Arrows indicate the transcription direction. Vertical colored bars indicate exon position. For *Pristis* and *Amblyraja* genomic mapping, exon position was located by BLASTing cDNA sequences of distant species, so they are putative. Gene colors follow the color code used in Figure 1. Position along the genomic scaffold or contig is indicated in base pair (gray numbering).

**FIGURE 3:**
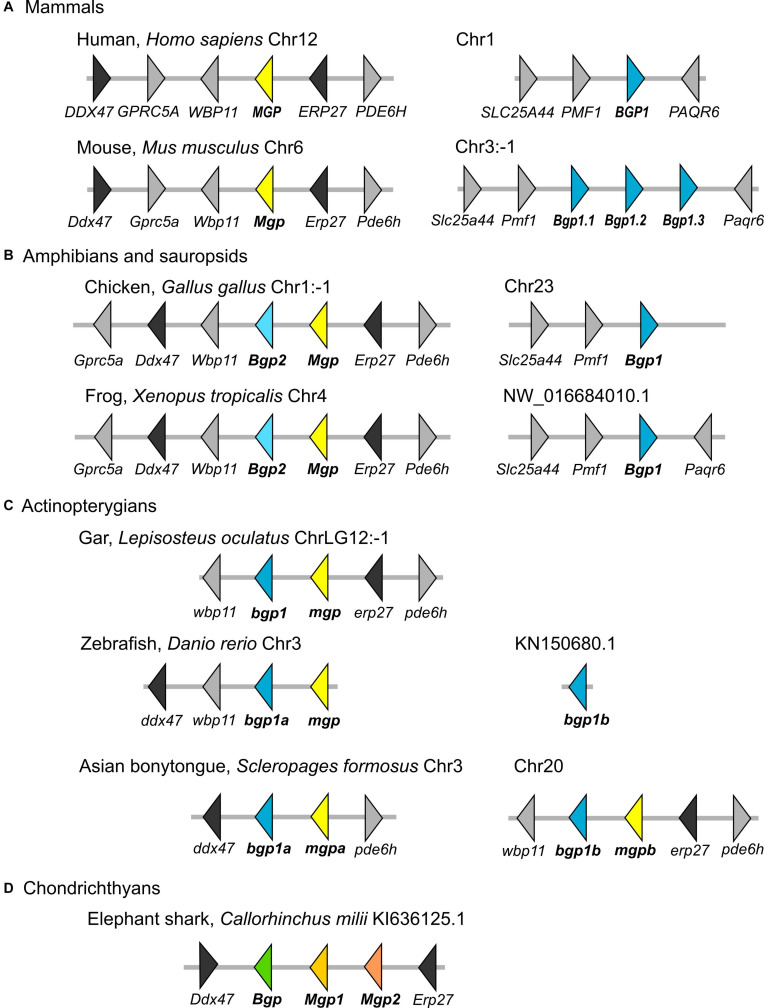
Genomic organization of the *Mgp/Bgp* gene clusters in reference osteichthyan genomes as annotated in currently available databases. **(A)** Two mammalian genomes with separated *Mgp* and *Bgp* loci; **(B)** Two non-mammalian tetrapod genomes with one *Bgp* locus, and one tandem *Bgp* and *Mgp* genes on homologous loci; **(C)** One non-teleost actinopterygian with only one locus where *Mgp* and *Bgp* genes are tandemly organized, and two teleost genomes with two loci, where *mgp* and *bgp* are tandemly organized or single. **(D)** The elephant shark as a representative of chondrichthyans. Several syntenic genes were selected to support the homology of the compared loci. Distance between genes is not to scale. Gene names and corresponding color refer to our phylogenetic analyses, and the correspondence to gene names in databases is found in the Supplementary Material 1.

All three coding sequences were predicted in the available elephant shark genome and all assigned to a single genomic contig (Figure 2) together with two genes bordering the syntenic regions, *Erp27* and *Ddx47*, as identified in other syntenic regions from bony fishes (see Figure 3). The identified cDNA sequences of *Mgp1*, *Mgp2*, and *Bgp* could be assigned to a single scaffold in the small-spotted catshark draft genome in synteny with *Ddx47* and *Erp27* (see Figure 2). In two batoid genomes (*Amblyraja* and *Pristis*), *Mgp2* and *Bgp* genes could be assigned to a single contig together with *Erp27* and *Ddx47*. However, the *Mgp1* gene was located on another scaffold in the *Amblyraja* genome, outside of the locus identified by the presence of *Erp27* and *Ddx47* (Figure 2), and no *Mgp1* gene could be identified in the *P. pectinata* genome.

The comparison to the genomic data in bony fishes was made in two steps. First, an overview of the genomic locations in tetrapods shows that in the human genome, there are separated loci for the *Mgp* (chromosome 12) and the *Bgp1* (BGLAP on chromosome 1) genes for which we highlighted the position of syntenic genes (Figure 3). All occurrences of the tetrapod *Bgp2* gene (as identified in our phylogenetic reconstruction) are in the *Mgp* locus in lissamphibians and sauropsids (Figure 3, and verified by BLAST on Ensembl available genomes of *P. sinensis*, *M. unicolor*, and *P. vitticeps*, not shown).

In a second step, we searched the homologous loci in actinopterygians (ray-finned fishes) outside of teleost fishes: the syntenic markers linked to *Bgp1* in tetrapods would not co-localize with any known sequence of either *Bgp* or *Mgp* in actinopterygians (Figure 3). The actinopterygian *Bgp1* gene was located in the *Mgp* locus, as defined by the presence of syntenic markers such as *Erp27* and *Ddx47* (Figure 3, and verified by BLAST on the available genome of *E. calabaricus*). This *Bgp1* copy is identified as *Bglap* or *Osteocalcin-like* (however, not annotated in the spotted gar) in the available databases (but see Supplementary Material 1 for predicted gene IDs). Within teleost fishes, the zebrafish *D. rerio* is usually used as a reference species, however, the contig where *bgp1b* is located is very short and does not give syntenic gene markers, while *bgp1a* and *mgp* are located close to each other on chromosome 3 (Figure 3). In Figure 3, we illustrate the genomic loci in the Asian bonytongue *S. formosus*, where each of the two teleost-specific copies of *bgp1* are found adjacent to one *mgp* gene that we named *mgpa* and *mgpb*, each of these genomic loci with either a sequence coding for *erp27* or *ddx47*, but both regions including a paralog of the *pde6h* gene. In all other teleost genomes that we have screened (see the sequences chosen for the phylogenetic reconstruction, and Supplementary Material 1), the *mgp* sequence was found in synteny with the *bgp1a* sequence, together with *ddx47*/*wbp11*, while the *bgp1b* sequence was found with *erp27* but without another copy of *mgp* (not shown).

### Protein Domains

**FIGURE 4:**
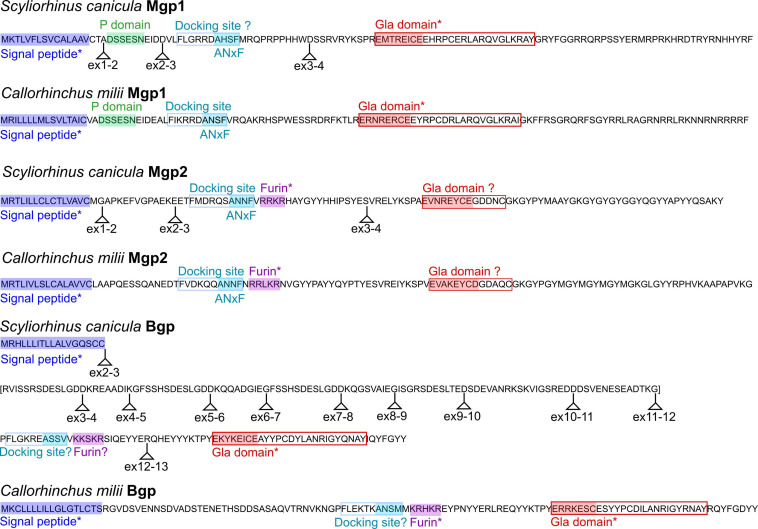
Conserved protein domains in the small-spotted catshark and the elephant shark Mgp/Bgp sequences. The small-spotted catshark Mgp/Bgp sequences are predicted from RNAseq, with location of exonic junctions (ex2–3: junction between exon 2 and exon 3); the elephant shark Mgp/Bgp sequences are predicted from genomic sequences (no exon junction showed). Domains predicted by InterPro, SMART, or FIMO are marked with an asterisk. Other domains are highlighted from their conserved alignment with previously characterized protein domains. Question marks are for domains identified after alignment but showing non-functional mutations. The small-spotted catshark Bgp sequence predicted from exons 3 to 11 is in bracket as it poorly aligns to any other vertebrate Bgp sequences.

The prediction of functional protein domains by InterproScan and SMART led to the recognition of a signal peptide for all sequences, but of a general Gla domain only in Bgp and Mgp1 proteins, excluding the Mgp2 sequences of the small-spotted catshark or elephant shark. The FIMO algorithm also identified a furin cleavage site in the Mgp2 sequence (see Figure 4). This was unexpected as it is typical for Bgp proteins but not of Mgp (Laizé et al., 2005). To further describe the presence, absence, and conservation of functional protein domains, we aligned characterized protein sequences of either Mgp or Bgp proteins (from human, mouse or chicken, and zebrafish) to those of the small-spotted catshark and elephant shark (Supplementary Material 6 and 7) and identified the expected location of specific functional domains of Mgp and Bgp proteins as previously described (Laizé et al., 2005).

The central motif for the Gla domain ExxxExC could be identified in the Mgp2, as well as in Mgp1 and Bgp sequences (Figure 4). However, the C-terminal part of the Gla domain was poorly aligned in the Mgp2 sequences, suggesting a divergent Gla domain in the Mgp2 paralog. In addition, no phosphorylation site could be identified in the Mgp2 sequences.

Mgp1 sequences (both from the small-spotted catshark and elephant shark) displayed well-conserved signal peptide, phosphorylation site, and general Gla domain (Figure 4). The expected ANxF site upstream to the Gla domain and supposed to participate in the docking site for the gamma-carboxylase (Viegas et al., 2013) was conserved in the elephant shark but modified to AHSF in the small spotted catshark questioning the functionality of this site (Figure 4).

In Bgp protein sequences, a signal peptide was also well conserved, followed by a furin cleavage site in the elephant shark that was not predicted in the small spotted catshark sequence because of a modification to KKSKR (Figure 4). A well-conserved Gla domain including the highly conserved Gla motif ExxxExC was present in the elephant shark and small-spotted catshark Bgp sequences.

By aligning each cDNA sequence with the genomic locus, we could map the exonic junctions on the full length protein sequences of the small-spotted catshark: *Mgp1* and *Mgp2* display conserved intron/exon structure [four exons, ATG and peptide signal coding sequence in the first exon, docking site coding sequence in the third exon, and Gla domain in the fourth exon: see Figure 4 and compare to bony fishes (Laizé et al., 2005; Viegas et al., 2013)]. On the other hand, the small-spotted catshark Bgp sequence displayed a divergent exon-intron structure [as compared to bony fishes (Laizé et al., 2005; Viegas et al., 2013)]: 13 exons and a series of imperfect repeat sequences between exons 3 and 12 (exons 3, 5, 7 code for very similar protein sequences), revealing important divergence of the gene structure. Because our cDNA sequence is reconstructed from RNAseq data, we cannot exclude the existence of splicing variants that would not include these extra-exons. Also, the elephant shark sequence does not include these repeated exons so they may be specific for the lesser spotted catshark (so a product of recent evolution).

### Gene Expression Patterns in the Embryonic Small-Spotted Catshark *Scyliorhinus canicula*

All three identified *Mgp*/*Bgp* sequences generated distinct expression patterns in the small-spotted catshark embryos by *in situ* hybridization. The selected stages of development were chosen in order to cover one time point before and another after the initiation of mineralization in the developing vertebrae (Enault et al., 2016) and during tooth development.

#### Mgp1 Expression

**FIGURE 5:**
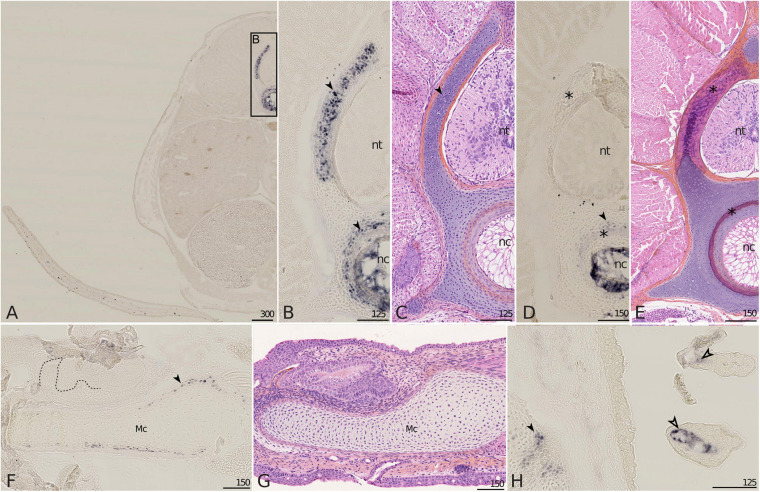
*Mgp1* gene expression on sections of late developing embryos of the small-spotted catshark *Scyliorhinus canicula*. **(A–C)** A total of 6 cm long embryos showing *Mgp1 in situ* hybridization, general **(A)** and closer **(B)** view on transverse sections at the level of the pectoral fin and Hematoxylin-Eosin-Saffron (HES) staining of a comparable zone to **B (C)**. **(D,E,H)** Transverse sections of 7.7 cm long embryos displaying *Mgp1 in situ* hybridization **(D,H)** or HES staining **(E)**. **(F)** *Mgp1 in situ* hybridization on a parasagittal section of the Meckel’s cartilage of a hatchling embryo with developing teeth [dotted line separates the epithelial (e) and mesenchymal (m) compartments of teeth]. **(G)** HES staining on a comparable zone to **(F)**. **(H)** Branchial basket with gills. *Mgp1* expression is detected in neural arch and vertebral body chondrocytes (filled arrowheads in **B**,**D**) before but not after mineralization (located with asterisks in **D**,**E**); in chondrocytes in the periphery of the Meckel’s cartilage before mineralization (filled arrowhead in **F**) and of other skeletal elements (filled arrowhead in **H**); in the connective tissue cells that surround vasculature in gills (open arrowhead in **H**). Mc, Meckel’s cartilage; nc, notochord; nt, neural tube. Scales are in μm.

In the 6 cm long embryo, the expression of *Mgp1* was detected in the developing vertebrae: in the cartilaginous core of neural arches, in a cartilaginous ring surrounding the notochord and also in notochordal cells (Figures 5A,B). At this stage, these zones of expression are not mineralized (Figure 5C; see Enault et al., 2016), but neural arches and the cartilage surrounding the notochord will show strong mineralization in embryos measuring 7.7 cm (Figure 5E; see Enault et al., 2016). On the 7.7 cm long embryo, the expression of *Mgp1* was no longer detected in neural arches, appeared faint in the cartilage surrounding the notochord, but was still strong in the notochord which is not a site of mineralization (Figure 5D). In the Meckel’s cartilage, the expression of *Mgp1* was not detected in developing teeth but was detected in a sub-perichondral population of chondrocytes (Figure 5F) at a time when no mineralization has started in the lower jaw cartilage (Figure 5G), but in a zone prefiguring the site of tesseral mineralization (Enault et al., 2015). Further expression in chondrocytes was detected in the pectoral girdle cartilages in a sub-perichondral layer of chondrocytes located in a contact zone between two cartilages (Figure 5H, filled arrowhead). Finally, expression of *Mgp1* was observed in gills, both in the endothelium of the vascular system and in undifferentiated mesenchyme surrounding vascularization (Figure 5H).

#### Mgp2 Expression

**FIGURE 6:**
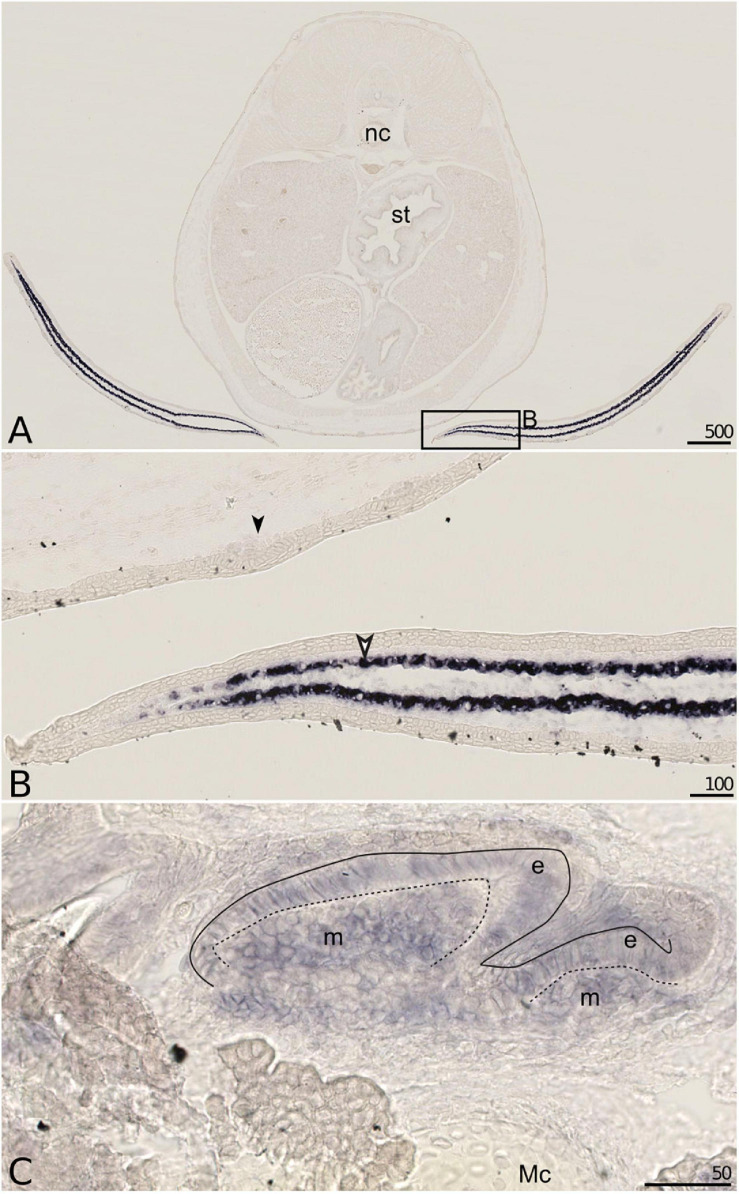
*Mgp2* gene expression on sections of late developing embryos of the small-spotted catshark *Scyliorhinus canicula*. **(A–C)** *Mgp2 in situ* hybridization: general **(A)** and closer **(B)** views on transverse sections of a 6 cm long embryo. **(C)** *Mgp2 in situ* hybridization on a parasagittal section of the Meckel’s cartilage (Mc) of a hatchling embryo with developing teeth (e, epithelial compartment; m, mesenchymal compartment of tooth buds; dotted line separates these two compartments). Expression detected in cells surrounding ceratotrichiae (open arrowhead in **B**), epithelial and mesenchymal cells of developing (unmineralized) tooth buds (in **C**). Mc, Meckel’s cartilage; nc, notochord; st, stomach. Scales are in μm.

The expression of the *Mgp2* gene in the small-spotted catshark was restricted and could be observed with very strong signal in the developing fins, in 6 (Figures 6A,B) and 7.7 cm (not shown) long embryos, in the mesenchymal tissue surrounding and most probably synthesizing ceratotrichiae, the semi-rigid fibers that make up the fin support in cartilaginous fishes. Weaker signal was detected in developing unmineralized tooth bud of the lower jaw (Figure 6C).

#### Bgp Expression

**FIGURE 7:**
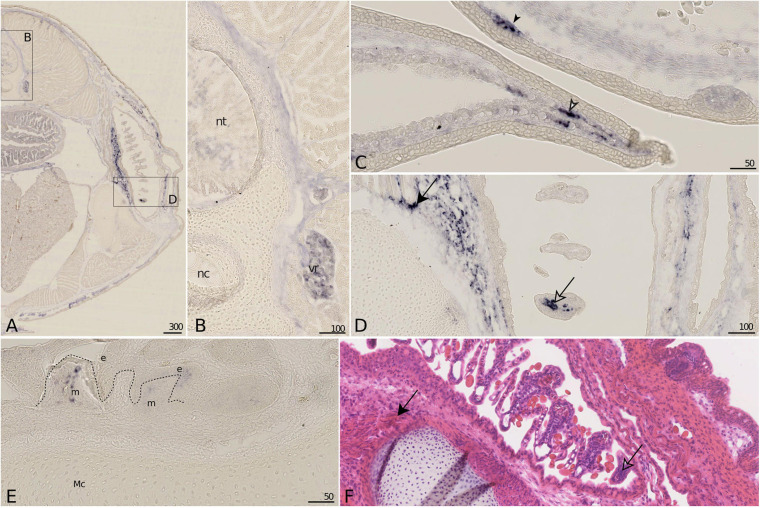
*Bgp* gene expression on sections of late developing embryos of the small-spotted catshark *Scyliorhinus canicula*. **(A–D)** *Bgp in situ* hybridization: general **(A)** and closer **(B–D)** views on transverse sections of a 7.7 cm long embryo. **(E)** *Bgp in situ* hybridization on a parasagittal section of the Meckel’s cartilage of a hatchling embryo with developing teeth [dotted line separates the epithelial (e) and mesenchymal (m) compartments of teeth]. **(F)** HES staining of a comparable zone to **(D)**. Expression detected in nerve root **(B)**, cells surrounding ceratotrichiae (open arrowhead in **C**), mesenchymal cells of scale placodes (filled arrowhead in **C**), and mesenchyme of mature (dentin deposition) tooth buds **(E)**, connective tissue at muscle attachment (black arrow in **D**,**F**) and at the tip of vasculature in gills (open arrow in **D**,**F**). Mc, Meckel’s cartilage; nc, notochord; nt, neural tube; vr, nerve root. Scales are in μm.

Bone Gla protein showed a widespread low-level expression in many connective tissues in the 7.7 cm long embryo (Figures 7A,B) but could not be detected in any chondrocyte population, neither in early (data not shown) or late stage vertebrae (Figure 7B) nor in Meckel’s cartilage (Figure 7E). Stronger detection of *Bgp* expression was observed in the cells of the nerve root (Figure 7B), the mesenchymal cells of scale buds at a placode stage (Figure 7C, filled arrowhead), mesenchymal cells in connective tissues surrounding muscles of the branchial apparatus with strong expression in the zone of attachment between muscle fibers and cartilaginous units (Figure 7D, black arrow), few mesenchymal cells of mineralized teeth (Figure 7E). Some weaker signal could be detected in the epithelium and mesenchyme of non-mineralized tooth buds (Figure 7E). *Bgp* expression could also be detected in gill tissues, restricted to the connective mesenchyme that surrounds the vascular system (open arrow), but its expression could not be observed in the vascular endothelium as seen with *Mgp1* (Figures 7D,F and compare with Figure 5H). Finally, *Bgp* expression was detected in cells of the pectoral fin tip, in the mesenchymal tissue surrounding ceratotrichia in 6 cm long (not shown) and 7.7 cm long embryos (Figure 7C, open arrowhead).

#### Embryonic Patterns of Expression

**FIGURE 8:**
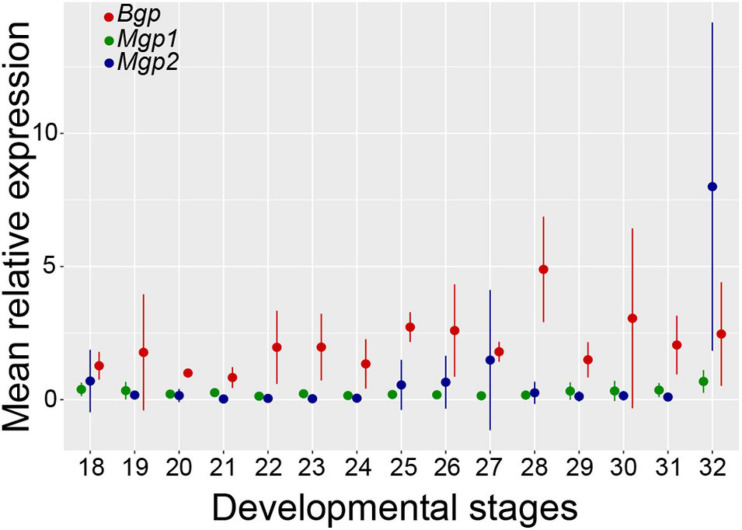
Relative levels of *Mgp1*, *Mgp2*, and *Bgp* mRNA expression in early embryos of the small-spotted catshark (stage 18–32). The value set to 1 was chosen as the *Bgp* mean value at stage 20; for each gene, at each developmental stage, mean values are represented with standard deviation. At each point, 3 < *n* < 4.

Total RNA extracts obtained from whole embryos of the small-spotted catshark from stage 18 (end of neurulation) to stage 32 (late organogenesis) (Ballard et al., 1993) allowed the evaluation of relative expression levels for *Bgp, Mgp1*, and *Mgp2* over the course of organogenesis in the small-spotted catshark (Figure 8). *Bgp* expression generally tended to be higher than the expression of the Mgp genes during the stages 18–32, to the exception of *Mgp2* expression at stage 32 (Figure 8). The results of the one-way ANOVA testing for gene expression variation over developmental stages were non-significant for the genes *Mgp1* and *Bgp*. However, the one-way ANOVA for the *Mgp2* gene indicated a difference between group means at the *P* < 0.1 threshold, probably due to the higher expression level observed at the stage 32. Stage 32 may be the stage of initiation of ceratotrichiae development (there is no sign of ceratotrichiae in pectoral or pelvic fins in stage 30 embryos in Tanaka et al., 2002) explaining the initiation of stronger expression at stage 32.

## Discussion

### An Evolutionary Scenario for Mgp/Bgp Gene Duplicates

**FIGURE 9:**
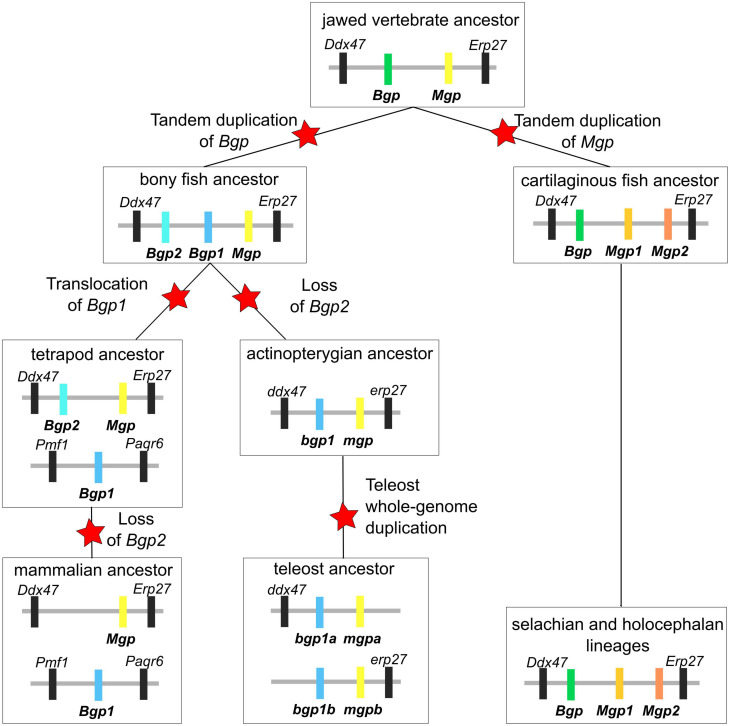
Evolutionary scenario integrating all synteny and phylogenetic data obtained in the cartilaginous fish clade. Each schematic summarizes the identity and location of each gene over the diversification of jawed vertebrates. Evolutionary events such as gene duplication, translocation and loss are marked with a red star. Genes are labeled with the same color code as in Figures 1, 2.

Syntenic and phylogenetic data gathered in this study allow drawing an evolutionary scenario for the genomic organization and diversification of the Mgp/Bgp gene family, under a most-parsimonious model of evolution (Figure 9 and Supplementary Material 4). In the bony fishes, our data allow testing previously proposed hypotheses. The phylogenetic relationships between Bgp1 and Bgp2 (Figure 1) suggest that these two copies emerged from a gene duplication in the last common ancestor of bony fishes which is congruent with previous identification and phylogenetic reconstruction including Bgp2 [previously named OC3 (Cancela et al., 2014)] where data from chondrichthyans were missing. This node (and others) still displays poor robustness when tested with SH-aLRT: these low values may be dependent on the little number of positions in our alignment (129 aa), a tendency which amplifies with higher number of protein sequences in the alignment and which cannot be easily corrected for, due to the small length of the studied proteins.

In addition, we show that a translocation of *Bgp1* most probably occurred in the sarcopterygian or tetrapod stem lineages, while *Bgp2* was lost convergently in actinopterygians and mammals (Figure 9). Unfortunately, no sequence homologous to Bgp could be identified in the available genomic databases of the coelacanth (NCBI or Ensembl, by TBLASTN search of the gar Bgp1 sequence), which could have helped in determining more precisely the timing of the *Bgp1* translocation. Finally, our phylogenetic reconstruction and teleost genome data-mining allowed the annotation of the previously named *OC2* gene (Cancela et al., 2014) as one of the two *bgp1* paralogs (Figures 1, 9) generated by the teleost-specific whole-genome duplication (Amores et al., 1998). We also identified two *mpg* co-orthologs in the Asian bonytongue genome, in tandem organization with each of the *bgp1a* and *bgp1b* copies, with two *pde6h* gene copies and located in synteny with either *erp27* or *ddx47* (Figure 3), supporting that the duplicated genes could have originated from the teleost-specific genome duplication. However, these two *mgp* copies were found only in one species within the genomes available in Ensembl: only one *mgp*, in synteny with *bgp1a*, is found in all other examined teleost genomes, which would imply a secondary loss of this *mgpb* gene duplicate in all examined taxa. As a consequence, further analysis of the genomic data in teleost fishes is still needed to support this scenario.

In the chondrichthyan lineage, we uncovered a specific tandem duplication in the Mgp locus leading to the *Mgp1* and *Mgp2* genes. Within chondrichthyans, an additional event of translocation may have occurred for the *Mgp1* copy in the batoid lineage (observed in *A. radiata*, Figure 2). However, because this is a single observation and because the *Amblyraja Mgp1* copy was identified in a short scaffold, we still cannot rule out the possibility of an assembly artifact. Additional genomic data from batoids are necessary to test the robustness of this observation.

Our results demonstrate that the location of the actinopterygian *Bgp1* and of the chondrichthyan *Bgp* is in the *Ddx47*/*Erp27* locus, which suggests an ancestral location of *Bgp* in this locus, later followed by tandem duplication that generated *Bgp1* and *Bgp2* in bony fishes. The ancestral *Mgp* and *Bgp* genes were, in this scenario, tandem duplicates in the last common ancestor of jawed vertebrates (Figure 9). No other closely related genes to Mgp/Bgp family have been reported for jawed vertebrates. In addition, no similar sequence was found in the genomic data of the lamprey (although the *Ddx47*/*Wbp11* locus can be identified on chromosome 3 of the kPetMar1 assembly), nor in Amphioxus nucleotide dataset (NCBI). Taken together, these three last arguments let us hypothesize that the evolution of Mgp/Bgp family cannot be explained by two rounds of whole genome duplications (Ohno, 1970), that occurred before the divergence of jawed vertebrates and resulted in expansion of many gene families from one to four genes located in different paralogons (Dehal and Boore, 2005). The inability to detect closely related gene families might be explained by several scenarios: (i) complete loss of other paralogs, ancestrally to jawed vertebrates [most frequently observed situation (Blomme et al., 2006)], (ii) rapid and extensive evolution of the coding sequences making sequence similarity searches inefficient, and (iii) *de novo* evolution of an ancestral *Bgp*/*Mgp* gene after the two rounds of genome duplication (Van Oss and Carvunis, 2019).

With the evolutionary scenario presented here, the orthology relationships between jawed vertebrate genes of the *Mgp/Bgp* family are more complex than usually considered, as the *Bgp* gene found in cartilaginous fishes is not a one-to-one ortholog to the *Bgp* copy (*Bgp1*) found in actinopterygian (non-teleost) fishes or in mammals. In addition, the *Bgp1* copy found in sarcopterygian genomes has gone through a translocation event that may have modified the transcriptional regulation, and therefore the function, of its orthologous copy in sarcopterygian fishes.

### Diversity of Expression Patterns in Cartilaginous Fishes and Functional Implications

Previous hypotheses accounting for the evolution of Mgp/Bgp sequences relied on partial sequence data and proposed that only a single *Mgp* gene was present in cartilaginous fishes (Cancela et al., 2014). The survey of transcriptomic and genomic data here reveals the presence of three genes, two *Mgp* genes and one *Bgp*. Few significant aspects of Mgp/Bgp gene evolution in chondrichthyans can be derived from the conservation of functional protein domains. Mgp1, as partly previously described in shark (Price et al., 1994; Rice et al., 1994; Ortiz-Delgado et al., 2006), displays well-conserved signal peptide, phosphorylation sites, carboxylase docking site, and a full Gla domain. This Gla domain is known to be able to bind calcium when secreted in the extracellular matrix and then acts as an inhibitor of mineralization under the condition that Mgp protein is phosphorylated (Schurgers et al., 2007). On the other hand, our data suggest the divergence of the Gla domain in the Mgp2 protein, although the core Gla motif was observed in our alignments, together with a loss of the phosphorylation domain that follows the signal peptide in other Mgp proteins (Figure 4). From these observations, we could expect Mgp1 to display a conserved Mgp function as known in bony fishes, while Mgp2 may have undergone partial or complete change of function. These observations suggest that after the *Mgp1*/*Mgp2* duplication event in cartilaginous fishes, the *Mgp2* copy underwent neofunctionalization, while *Mgp1* kept the ancestral function (Ohno, 1970). The chondrichthyan Bgp preprotein as described from transcriptomic data of the small-spotted catshark shows conserved signal peptide, followed by a long stretch of non-conserved amino-acids that partly originate from repeated sequences through addition of new exons (Figure 4). No functional protein domain was predicted in this zone of the protein. It was followed by a putative docking site, and then a better conserved C-terminal sequence including a well conserved Gla domain (Figure 4). In the elephant shark, a furin site is conserved in the N-terminal side of the Gla domain. Provided the cleavage site is indeed functional, the mature Bgp protein would then be very similar to Bgp in bony vertebrates: cleavage facilitates carboxylation and as a consequence the affinity of Bgp for hydroxyapatite (Al Rifai et al., 2017).

We also questioned the function of chondrichthyans *Mgp1*, *Mgp2*, and *Bgp* through the survey of their expression patterns in the small-spotted catshark. Some observed expression patterns are shared with bony vertebrates (Table 1), but some appear to be specific to cartilaginous fishes. Of course, *in situ* hybridization data show which cells express which genes, but do not help in determining if the protein is produced, where and how much of it is secreted, nor what is the gamma-carboxylation status of the Mgp/Bgp proteins. Further proteomic studies would be necessary to resolve the protein status in terms of post-translational cleavage and carboxylation. As a result, the following discussion only speculates on the functional implications of gene expression patterns in the small-spotted catshark.

**TABLE 1:** Described expression of *Bgp* and *Mgp* genes in selected tissues and selected species of jawed vertebrates compared to data obtained for the small-spotted catshark (this study).

	Small-spotted catshark			Teleost fishes		Xenopus		Mammals		Chicken	
	Mgp1	Mgp2	Bgp	mgp	bgp1a	Mgp	Bgp1	Mgp	Bgp1	Mgp	Bgp1
Embryonic	+	+	+		+ (3)			+ (17)		+ (8)	
Chord	+	−	−		+ (3)					+ (8)	
Chondrocyte	+	−	−	+ (1)	−	+ (10)	− (10)	+ (11)	− (14)	+ (18)	− (12)
Chondrocyte (mineralized matrix)	−	−	−	+ (1)	+ (1)	+ (10)	− (10)	+ (11)	− (14)	+ (18)	+ (12)
Osteoblasts	na	na	na	− (1)	+ (1, 5)	− (10)	+(10)	+ (6)	+ (14)	− (18)	+ (12)
Early tooth/scale bud	−	+	+		+ (15) regeneration				− (13)	na	na
Late (mineralized) tooth/scale	−	−	+	+ (4)	+ (4)				+ (13)	na	na
Muscle and its connective tissue	−	−	+	+ heart (2, 5)	+ heart (5)	− (7)				+ (19)	
Vasculature and its connective tissue	+	−	+ gills	+ heart (2, 5)	+ arteries(5)			+ (11, 16)	+ (16)	+ (8, 18)	
Nerve root	−	−	+						+ (9)		
Ceratotrichia	−	+	+	−	− (1)	na	na	na	na	na	na

*Sources: (1) (Gavaia et al., 2006); (2) (Simes et al., 2003); (3) (Bensimon-Brito et al., 2012); (4) (Ortiz-Delgado et al., 2005); (5) (Viegas et al., 2013); (6) (Coen et al., 2009); (7) (Cancela et al., 2001); (8) (Correia et al., 2016); (9) (Ichikawa and Sugimoto, 2002); (10) (Espinoza et al., 2010); (11) (Luo et al., 1997); (12) (Neugebauer et al., 1995); (13) (Bleicher et al., 1999); (14) (Sommer et al., 1996); (15) (Iimura et al., 2012); (16) (Hao et al., 2004); (17) (Gilbert and Rannels, 2004); (18) (Dan et al., 2012); (19) (Wiedemann et al., 1998). na, not applicable (the anatomical structure does not exist in the specified taxon).*

A specific site of expression in the small-spotted catshark was found with a very restricted site of expression of *Mgp2* in the developing ceratotrichiae of the pectoral fin, also observed for *Bgp*. These shark ceratotrichiae are massive collagenous fibers that support the distal fin (Kemp, 1977) without being mineralized. Similar collagen-based fibrils named actinotrichia are found in teleost fishes (Durán et al., 2011), and together with lepidotrichiae (bony hemi-segments), they build up the typical fin rays found in actinopterygians fishes. These collagen-based fibers are supposed to be homologous between cartilaginous and actinopterygian fishes (Zhang et al., 2010). To our knowledge, the expression of *Mgp* or *Bgp1* has never been recorded in fish actinotrichia, although the expression of *Bgp1* was detected in the dermal bone of fin rays in several teleost fishes (Stavri and Zarnescu, 2013; Viegas et al., 2013) and in the cartilaginous supports of fins (Gavaia et al., 2006). These data therefore suggest a cartilaginous fish specific site of expression for *Mgp2* and *Bgp* in developing ceratotrichiae, be it an evolutionary innovation in this lineage, or a consequence of secondary loss of this site of expression in bony fish. The fact that this is a shared zone of expression between *Mgp2* and *Bgp* could support the hypothesis of an ancestral feature (that evolved before the duplication of the gene ancestral to *Bgp* and *Mgp*) or a secondary (chondrichthyan-specific) recruitment of both genes that may share regulatory elements, given their genomic proximity. This strong expression in fin ceratotrichiae, that are not mineralized structures, is not congruent with a mineralization function of Bgp proteins in the small-spotted catshark ceratotrichiae.

The remaining range of tissue with expression of the *Bgp* gene in the small-spotted catshark is not congruent either with the hypothesis of a function in the activation of mineralization: it is expressed in several soft tissues such as connective tissues surrounding muscles, nerve root and vasculature of the gills (Figure 7). These sites of expression were previously identified in tetrapods and actinopterygian fishes for both *Mgp* and *Bgp* genes (see Table 1 and discussion below). In most of these soft tissues, the expression of *Mgp* is considered to ensure inhibition of mineralization, but the function of *Bgp* in these tissues is still poorly understood. In the small-spotted catshark, the only site of *Bgp* expression that correlates with tissue mineralization is in the pulp of mineralized teeth, which is similar with other observations in tetrapods and teleost fishes (see Table 1 for references) but may be linked to non-mineralizing cells in the dental pulp, e.g., vascular system or innervation.

Finally, only the expression of *Mgp1* is strongly linked to the dynamic of skeletal mineralization in the small-spotted catshark: it is found expressed in subpopulations of chondrocytes that are specifically pre-mineralization chondrocytes (before areolar mineralization surrounding the notochord; before the initiation of tesserae mineralization; before globular mineralization in the neural arch (Debiais-Thibaud, 2019); and the expression goes down at the time when mineralization initiates. *Mgp1* is also expressed in the cells of the notochord that never mineralizes. These observations are more congruent with a function of *Mgp1* in the inhibition of mineralization during the maturation of the skeletal tissues in the small-spotted catshark.

### Comparative Analyses and the Evolution of Mgp and Bgp Functions

There is currently no possibility to compare the two *Bgp* copies in tetrapods because expression data have been described only for the *Bgp1* copy in the chicken and the tropical clawed frog, and we did not find any description of *Bgp2* expression (see references in Table 1).

The early and strong embryonic expression detected for *Bgp* in the small-spotted catshark is reminiscent of other studies showing an early expression of *Bgp1* in the zebrafish (Bensimon-Brito et al., 2012), although others detected neither embryonic nor early larval expression of *Bgp1* in other teleost fish (Pinto et al., 2001). On the other hand, *Mgp* genes are also expressed in early embryos: in the vascular system of the avian embryo (Correia et al., 2016) and developing limbs and lungs of the mouse as early as E10.5 (Luo et al., 1995; Gilbert and Rannels, 2004). All these data suggest a shared and ancestral function of Mgp and Bgp proteins during embryogenesis, before tissue and cell differentiation. A function in inhibitory interaction with Bmp proteins was shown for the Mgp protein in human cells (Zebboudj et al., 2002) as well as with the transforming growth factor-β pathway (Oh et al., 2000) which may explain an early expression during morphogenesis. Another conserved aspect of Mgp and Bgp genes is their expression in the tissues surrounding certain muscles and the vasculature along the embryonic and adult period. This zone of expression is shared between *Mgp1* and *Bgp* in the small-spotted catshark, similar to previous descriptions in the zebrafish and mammals (Hao et al., 2004; Simes et al., 2004; Viegas et al., 2013). In these sites of expression, it is accepted that Mgp and Bgp proteins act as mineralization inhibitors, by interacting with the BMP pathway (Yao et al., 2006) or by their properties in their uncarboxylated forms (Schurgers et al., 2005, 2007; Zoch et al., 2016). These two properties might be ancestral characteristics for both Mgp and Bgp in jawed vertebrates, and of the ancestral gene that gave rise to Mgp and Bgp by duplication.

Finally, the expression of the small-spotted catshark *Bgp* in the nerve root is also a characteristic previously described in the mouse (Ichikawa and Sugimoto, 2002) and therefore suggests an ancestral role of the *Bgp* copy in the nervous system of jawed vertebrates. The function of Bgp in the nervous system has not been fully uncovered but it has been proposed to be an active neuropeptide in sensory ganglia (Patterson-Buckendahl et al., 2012).

We previously concluded on the putative function of Mgp1 in the inhibition of mineralization during the maturation of the skeletal tissues in the small-spotted catshark. This observation is shared with all described gene expression patterns in skeletal tissues in other jawed vertebrates. As a consequence, it supports the hypothesis of an ancestral involvement of the Mgp/Bgp gene family in the regulation of skeletal mineralization, although limited to the negative regulation of calcium deposition in the cartilage by members of the *Mgp* clade.

## Concluding Remarks

The description of the *Mgp/Bgp* complement in cartilaginous fishes reveals complex dynamic evolution of this gene family during jawed vertebrate evolution. Although previously reported expression of *Mgp* and *Bgp1* in tetrapods was found involved in the regulation of mineralization in skeletal tissues, only *Mgp1* displays association with skeletal tissue differentiation in the small-spotted catshark embryo, and its expression pattern is congruent with an ability to inhibit mineralization in the step preceding precipitation of calcium in the cartilaginous matrix. The ability to activate mineralization in skeletal tissues may finally be a specificity of the *Bgp1* bony fish copy: either because it evolved after the divergence with cartilaginous fishes or because cartilaginous fishes have secondarily lost bone-associated genetic toolkits as they lost bone tissues (Donoghue et al., 2006; Brazeau et al., 2020).

## Data Availability Statement

The original contributions presented in this study are included in the article and Supplementary Material, further inquiries can be directed to the corresponding authors.

## Ethics Statement

Handling of small-spotted catshark embryos followed all institutional, national, and international guidelines (European Communities Council Directive of 22 September 2010 [2010/63/UE]): no further approval by an ethics committee was necessary as the biological material is embryonic and no live experimental procedures were carried out.

## Author Contributions

MD-T and TH designed the study, analyzed the data, and drafted the manuscript. NL performed the synteny and phylogenetic analyses. CM-M generated qPCR expression data. SV performed the *in situ* expression experiments. TH performed the genome data mining. All authors contributed to the article and approved the submitted version.

## Conflict of Interest

The authors declare that the research was conducted in the absence of any commercial or financial relationships that could be construed as a potential conflict of interest.

## Footnotes

1 http://skatebase.org/

## Supplementary Material

Supplementary Material 1 References of all sequences used in this manuscript and extracted from open sequence databases or from locally assembled transcriptome data.

Supplementary Material 2 Protein sequence alignment generated by MAFFT.

Supplementary Material 3 Protein sequence alignment generated by HmmCleaner automatic cleaning and used to produce the phylogenetic tree reconstruction.

Supplementary Material 4 Contracted gene and species trees used with Treerecs and the visual output for the maximum parsimony scenario.

Supplementary Material 5 Unrooted tree obtained after the phylogenetic reconstruction. Colors and gene clade annotations are as in Figure 1.

Supplementary Material 6 Alignment identifying conserved protein regions of Bgp proteins between the human *Homo sapiens*, the chicken *Gallus gallus*, the zebrafish *Danio rerio*, the small spotted catshark *Scyliorhinus canicula*, and the elephant shark *Callorhinchus milii*: signal peptide in the first 22 amino-acids, furin cleavage site in positions 162–166 of the alignment, core Gla domain starting on position 186.

Supplementary Material 7 Alignment identifying conserved protein regions of Mgp proteins between the human *Homo sapiens*, the zebrafish *Danio rerio*, and both Mgp1 and Mgp2 sequences found in chondrichthyans. Signal peptide is found in the first 20 amino-acids, conserved phosphorylation domain in the Mgp1 sequences in positions 28–36, a partially conserved ANxF domain located in position 48–51 with a F in position 42 (considered as a carboxylase docking site), core Gla domain starting on position 83 with poor conservation of the Mgp2 sequence after position 90. Note the furin cleavage site in positions 53–56 of the alignment, found in *Callorhinchus milii* Mgp2 sequence only.
