# Supplementary material for: Evolution of Matrix Gla and Bone Gla Protein Genes in Jawed Vertebrates
Source: Front Genet. 2021 Mar 10;12:620659. doi: 10.3389/fgene.2021.620659 (PMC8006282; doi:10.3389/fgene.2021.620659)
Supplement: Supplementary Material 2 — Protein sequence alignment generated by MAFFT. [file Data_Sheet_2.DOCX]

%Supplementary Material 2. Protein sequence alignment generated by MAFFT (delete this line to get the fasta format file)

>Anolis-carolinensis-Mgp

------------------------------------------------------------

-----------------MRT-LII-LALLAVLMMA--AF----CY---------------

------------------------------------------------------------

------------------------------------------------------------

--------------------------ESHE-----------------SIES---------

------------HELT---------SPFINR-RHASIFM-RTPPENRRNP---YLYE---

------R--IRG-RNKSTQERQREACEDYYPCDTYALRYGYAAAYKRFFG-----QRRGK

------------------------------------------------------------

------------------------------------------

>Pogona-vitticeps-Mgp

------------------------------------------------------------

-----------------MRT-LII-LALLAVLMMT--AY----CY---------------

------------------------------------------------------------

------------------------------------------------------------

--------------------------ESHE-----------------SIES---------

------------HEFT---------SPFINR-RHANIFM-RTPPEDRRDH---YMRE---

------R--IRE-RSKSPQELQRERCEEYIPCERYAMRHGYVAAYKRYFG-----QRRGK

------------------------------------------------------------

------------------------------------------

>Chrysemys-picta-belli-Mgp

------------------------------------------------------------

-----------------MRT-LIL-LTFLAVLMVA--AF----CY---------------

------------------------------------------------------------

------------------------------------------------------------

--------------------------ESHE-----------------SMES---------

------------HEFV---------NPFINR-RNANDFM-RAQP--RRNF---IVQE---

------R--IRE-RNKTPQERQREICEDYNPCERYAMRHGYIAAYKRYFG-----QQRGK

FE----------------------------------------------------------

------------------------------------------

>Alligator-sinensis-Mgp

------------------------------------------------------------

-----------------MRT-III-LALLAVLVMA--AF----CY---------------

------------------------------------------------------------

------------------------------------------------------------

--------------------------ESQE-----------------SMES---------

------------HEFT---------SPFVTR-QSAHDFM-RPDP--RQKA---IMRE---

------R--IRE-RHKSPQERHREICEDYYPCERYAYHHGYAAAYKRFFV-----KRTTK

------------------------------------------------------------

------------------------------------------

>Notechis-scutatus-Mgp

------------------------------------------------------------

-----------------MDG----------------------------------------

------------------------------------------------------------

------------------------------------------------------------

--------------------------GAHK------------------------------

---------------------------------------------NRRNF---FWQE---

------R--IRE-RRKTTQELQREECEDYSPCERYAMRHGYVAAYKRFFG-----QRRGE

------------------------------------------------------------

------------------------------------------

>Gallus-gallus-Mgp

------------------------------------------------------------

-----------------MRA-LIV-LVLLAVLVMA--AT----CY---------------

------------------------------------------------------------

------------------------------------------------------------

--------------------------ESHE-----------------SMES---------

------------HEYL---------NPFLNR-QRANGFI-RDDT--GLRA---VLQE---

------R--IRE-RNKAPQERQREICEDFHLCEQYALNHGYPAAYRHYFG-----RRRNK

------------------------------------------------------------

------------------------------------------

>Apteryx-owenii-Mgp

------------------------------------------------------------

-----------------MRT-LIV-LTLLAFLVMT--AT----CY---------------

------------------------------------------------------------

------------------------------------------------------------

--------------------------VSMY-----------------SEQS---------

------------LAFS---------DPFINR-RRANDFI-QPDM--RLQA---ISQE---

------R--IRE-RSKAPREHQREMCEDYYPCELYAYRHGYAAAYRHYFG-----RRRAK

------------------------------------------------------------

------------------------------------------

>Xenopus-tropicalis-Mgp

------------------------------------------------------------

-----------------MKT-LPV-ILLLALVAVV--AL----AY---------------

------------------------------------------------------------

------------------------------------------------------------

--------------------------DSYE-----------------SHES---------

------------LEVY---------DPFLNS-RKANSFM-NSQA--KNQ----RMNE---

------R--IRE-RNKSPRERQREACEDYDPCERYALRYGFSAAYKRYFG-----QRRGE

KK----------------------------------------------------------

------------------------------------------

>Xenopus-laevis-Mgp

------------------------------------------------------------

-----------------MKT-LPV-ILLLALAAAV--AF----AY---------------

------------------------------------------------------------

------------------------------------------------------------

--------------------------DSYE-----------------SHES---------

------------LEVY---------DPFLNS-RKANSFM-NSQA--KNQ----RMNE---

------R--IRE-RNKSPRERQREACEDYDPCERYALRYGFTAAYKRYFG-----QRRGE

KK----------------------------------------------------------

------------------------------------------

>Nanorana-parkeri-Mgp

------------------------------------------------------------

-----------------MKT-LGV-LLVLALAAVV--TL----AY---------------

------------------------------------------------------------

------------------------------------------------------------

--------------------------VRDE-----------------SRES---------

------------LEV----------NPFVSS-RTANTFM-SSQH--RTS----KMNE---

------R--IRE-RNKSPQERQREVCEDYKPCDRYALHHGYTAAYSRYFG-----GRTRG

K-----------------------------------------------------------

------------------------------------------

>Microcaecilia-unicolor-Mgp

------------------------------------------------------------

-----------------MRV-LFI-VALLAILAVV--TF----CS---------------

------------------------------------------------------------

------------------------------------------------------------

--------------------------DSHE-----------------SYES---------

------------FERY---------HPFVNR-RKANNFI-GLQQ--KKA----RTYE---

------R--IRE-LNKSPKERQREICEDHDLCELYAMRHGFQKAYKRYFG-----QKYGR

GK----------------------------------------------------------

------------------------------------------

>Monodelphis-domestica-Mgp

------------------------------------------------------------

-----------------MKT-LLF-ITLLAALAVI--AL----CY---------------

------------------------------------------------------------

------------------------------------------------------------

--------------------------ESHE-----------------SMES---------

------------YEM----------TPFINR-RKANNFM-SPQQ--RWRA---KHQE---

------R--VRE-RTKPTHEIHREACDDYTLCSRYAFIHGYSAAYNRYFR-----QQRNI

------------------------------------------------------------

------------------------------------------

>Felis-catus-Mgp

------------------------------------------------------------

-----------------MKS-LLL-LSILAALAVA--VL----CY---------------

------------------------------------------------------------

------------------------------------------------------------

--------------------------ESHE-----------------SMES---------

------------YEI----------YPFTNR-RNANTFI-SPQQ--RWRA---KAHE---

------R--IRE-RTKPTYEINREACDDFKLCERYAMVYGYNAAYNRYFQ-----QRRGG

K-----------------------------------------------------------

------------------------------------------

>Homo-sapiens-Mgp

------------------------------------------------------------

-----------------MKS-LIL-LAILAALAVV--TL----CY---------------

------------------------------------------------------------

------------------------------------------------------------

--------------------------ESHE-----------------SMES---------

------------YEL----------NPFINR-RNANTFI-SPQQ--RWRA---KVQE---

------R--IRE-RSKPVHELNREACDDYRLCERYAMVYGYNAAYNRYFR-----KRRGT

K-----------------------------------------------------------

------------------------------------------

>Mus-musculus-Mgp

------------------------------------------------------------

-----------------MKS-LLP-LAILAALAVA--TL----CY---------------

------------------------------------------------------------

------------------------------------------------------------

--------------------------ESHE-----------------SMES---------

------------YEI----------SPFINR-RNANTFM-SPQQ--RWRA---KAQK---

------R--VQE-RNKPAYEINREACDDYKLCERYAMVYGYNAAYNRYFR-----QRRGA

KY----------------------------------------------------------

------------------------------------------

>Loxodonta-africana-Mgp

------------------------------------------------------------

-----------------MKS-LLL-LTILAAFTVA--IL----CY---------------

------------------------------------------------------------

------------------------------------------------------------

--------------------------ESYE-----------------SLES---------

------------HEL----------SPFLNR-RYANSFI-SPQE--RWRA---KAQE---

------R--VRE-LRKPAHELNREACEDFGICERYAMLYGYNAAYNRFFR-----QRRGT

------------------------------------------------------------

------------------------------------------

>Ornithorhynchus-anatinus-Mgp

------------------------------------------------------------

-----------------MKT-LLL-LSLLVVLAAA--AV---GGY---------------

------------------------------------------------------------

------------------------------------------------------------

--------------------------ESHE-----------------SMES---------

------------YEM----------NPFIPR-RNANSFI-APRQ--RWRS---RAQE---

------R--ARE-LSKPSHEIQREACDDYPLCQRYARNYGYRAAYTRYPG-----GFRV-

------------------------------------------------------------

------------------------------------------

>Acipenser-naccarii-Mgp

------------------------------------------------------------

-----------------MRF-LIW-FAVVALWIAV--CF----SY---------------

------------------------------------------------------------

------------------------------------------------------------

--------------------------ESDE-----------------SFDS---------

------------GE-----------DVFMNP-YSANSFM-NSNTNRRQTG---YYYE---

------R--LME-RYKSPRERQRESCEEYTPCDRFARRYGYQQA---GIG-----RS---

------------------------------------------------------------

------------------------------------------

>Erpetoichthys-calabaricus-Mgp

------------------------------------------------------------

-----------------MQT-LLL-ATLLSLLAAI--TF----CY---------------

------------------------------------------------------------

------------------------------------------------------------

--------------------------DSHE-----------------SIES---------

------------RE-----------EIFMKP-YQANSFM-NRFN----------YRQ---

------R--LLN-AYKRPFERQREICEDFGPCENYARFRGYNRAYQYFFG-----KPGQT

TRN---------------------------------------------------------

----------------------------------------RY

>Anolis-carolinensis-Bgp2

------------------------------------------------------------

-----------------MEN-LLMVLLFAMVLLTA-PCC----CE---------------

------------------------------------------------------------

------------------------------------------------------------

-------------------------ADTNR-----------------AID----------

-------------E-----------GIKIKR-EVASAFV-RRQK--RSY----PYYE---

------R--YYE-RFKSPMEMKQEQCENYAPCDYYSEVVGFHAAYSHFFG-----HV---

------------------------------------------------------------

------------------------------------------

>Pogona-vitticeps-Bgp2

------------------------------------------------------------

-----------------MRE-IFLILLLLSLVLTN-PCC----CE---------------

------------------------------------------------------------

------------------------------------------------------------

-------------------------RDMDR-----------------ALD----------

-------------K-----------GVKINR-ETANAFV-RRQK--RSY----PYYE---

------R--YYE-MFKSPMELKHEQCESYAPCDYYAEVVGFPVAYRHFFG-----SA---

------------------------------------------------------------

------------------------------------------

>Notechis-scutatus-Bgp2

------------------------------------------------------------

-----------------MN--LLM-FLMFALLLTV-PCC----CE---------------

------------------------------------------------------------

------------------------------------------------------------

-------------------------GASSN-----------------ALD----------

-------------E-----------GIKIDQ-EKANTFI-RRQK--RAY----PYSE---

------R--YYE-MFKSPMEMRQEQCEHYAPCNYYSEIVGFPTAYRHYFG-----SI---

------------------------------------------------------------

------------------------------------------

>Chrysemys-picta-belli-Bgp2

------------------------------------------------------------

-----------------MRK-LLV-LLLLTLALAA-FCY----CE---------------

------------------------------------------------------------

------------------------------------------------------------

-------------------------RDSKD-----------------TLESH-----G--

------------VE-----------GVKVKR-ETANAFV-RRQK--RSY----PYYE---

------R--YYE-MYKSPMELRKEQCENYAPCDYLSDHVGFHAAYQRYFG-----RF---

------------------------------------------------------------

------------------------------------------

>Apteryx-owenii-Bgp2

------------------------------------------------------------

-----------------MRS-LLA-PLILALALAA-LCC----CE---------------

------------------------------------------------------------

------------------------------------------------------------

-------------------------------------------------KGK-----G--

------------MS-----------PLGIKR-EVANAFV-RRQK--RS-----DLYE---

------W--YFE-YYKSPMEQMHERCENYPPCDFLSDQIGFSMAYNRFFG-----RY---

------------------------------------------------------------

------------------------------------------

>Gallus-gallus-Bgp2

------------------------------------------------------------

-----------------MRK-LLA-PLILTLALAV-HCC----CE---------------

------------------------------------------------------------

------------------------------------------------------------

-------------------------KDPKE-----------------PSGSP-----S--

------------AA-----------SITVEK-EVANAFV-KRQK--RF-----DMYE---

------W--YSE-YYKSPMEQMRERCESYPPCDYLSEQIGFPMAYNRFFG-----RY---

------------------------------------------------------------

------------------------------------------

>Rhinatrema-bivittatum-Bgp2

------------------------------------------------------------

-----------------MRV-LMV--LLALGLAVL--------CD---------------

------------------------------------------------------------

------------------------------------------------------------

-------------------------SDSDSSYSASSNSTEESSSAHSSSESH-----S--

------------NEV----------AIKIRR-NTANALVNKRLK--RSY----DYYE---

------R--YYE-RFKSPLEMKKEQCENYWPCDYLSNQVGFYQAYRRYFG-----PV---

------------------------------------------------------------

------------------------------------------

>Microcaecilia-unicolor-Bgp2

------------------------------------------------------------

-----------------MRA-LIV--LIILGLAVL--CI----CG---------------

------------------------------------------------------------

------------------------------------------------------------

-------------------------SDSDSSYS-TSNSTEDFSSAHSPSESH-----S--

------------NEA----------VIKMRR-NTANSLVKKRQK--RSY----GYYE---

------W--IHE-QFKSPMEMKKEQCEEYWPCDYLSRQVGFHQAYRRYFG-----PV---

------------------------------------------------------------

------------------------------------------

>Xenopus-tropicalis-Bgp2

------------------------------------------------------------

-----------------MKE-LVI--LSLLVLSVY--SV----LH---------------

-------------KD---------------------------------------------

------------------------------------------------------------

-------------------------VKPMD-----------------SRKKEF-------

------------PE-----------DVKVTR-QAAHAII-KRVR--RGY----NYYE---

------R--YFP-RVKSPLELKKEQCENYSPCDQLSEWVGFYQAYQTYFG-----PV---

------------------------------------------------------------

------------------------------------------

>Xenopus-laevis-Bgp2

------------------------------------------------------------

-----------------MKE-LVI--LSLLVLSVY--SL----LH---------------

-------------KD---------------------------------------------

------------------------------------------------------------

-------------------------VKPMD-----------------STKKEF-------

------------PE-----------DVKVTR-QAAHAVI-KRVR--RGY----NYYE---

------R--YFA-RMKSPLELKKEQCENYLPCDQLSEWVGFYQAYQKYFG-----PV---

------------------------------------------------------------

------------------------------------------

>Pristis-pectinata-Bgp

------------------------------------------------------------

------------------------------------------------------------

------------------------------------------------------------

------------------------------------------------------------

------------------------------------------------------------

--------------------------PFLEK-QKANSVM-KRPR--RSI-----------

------------------------ICESYYPCDYLANRIGFQTAYQQYFG-----NY---

------------------------------------------------------------

------------------------------------------

>Amblyraja-radiata-Bgp

------------------------------------------------------------

------------------------------------------------------------

------------------------------------------------------------

------------------------------------------------------------

------------------------------------------------------------

--------------------------PFLGK-QKSDSVV-KRPG--RSI-----------

------------------------LCESFSPCHHLANRVGYQNAYQQYFG----------

------------------------------------------------------------

------------------------------------------

>Scyliorhinus-canicula-Bgp

ML--------------------------------------------ELLQLNNHRT----

--PSLGTRSFQQQGQVKMRH-LLL--ITLLALVGQ-SCCRVISSRSD--------ESLGD

DKREA--------ADIKG----------F---------SSHSDESL-------GDDKQQA

DGIEG----------------FSSHSDESLGDDKQGSV-----------------AIEG-

--------ISGRSD----E---SLTEDSDEVA---------------NRKSKVIG--SRE

DDDSVENESEADTK-----------GPFLGK-REASSVV-KKSK--RSIQ---EYYE---

------R--QHEYYYKTPYEKYKEICEAYYPCDYLANRIGYQNAYIQYFG-----YY---

------------------------------------------------------------

------------------------------------------

>Rhincodon-typus-Bgp

------------------------------------------------------------

------------------------------------------------------------

------------------------------------------------------------

------------------------------------------------------------

------------------------------------------------------------

-------------------------DPFLEK-HKADSVV-KRSR--RSIQ---EYYE---

------R--HHEYYYKTPYEKRREICESYYPCNYLANRIGFQSAYIQYFG-----YY---

------------------------------------------------------------

------------------------------------------

>Callorhinchus-milii-Bgp

------------------------------------------------------------

-----------------MKC-LLL--LILLGLGTL--CT----SRGV-------------

------------------------------------------------------------

------------------------------------------------------------

--------------------------DSVE---NNSDVAD-------STENE-----THS

DDSASAQVTRNVKN-----------GPFLEK-TKANSMM-KRHK--REYP---NYYE---

------R--LREQYYKTPYERRKESCESYYPCDILANRIGYRNAYRQYFG-----DY---

------------------------------------------------------------

-----------------------------------------Y

>Anabas-testudineus-mgpa

------------------------------------------------------------

-----------------MRS-LLQ-FLALCAAAAL--CV----CY---------------

------------------------------------------------------------

------------------------------------------------------------

--------------------------DSHE-----------------STES---------

------------DE-----------DLFVPA-NRANSFI-RPQR--RN-----EYNPVR-

-GFNYYD--LMR-KIKSAAERRSETCEDFRPCRLYSFQVGRQQAYNRYFG-----AQNQP

Q-----------------------------------------------------------

---------------------------------RPAGIR-RY

>Gasterosteus-aculeatus-mgpa

------------------------------------------------------------

-----------------MRS-LLQ-LLALCAAASF--CV----CY---------------

------------------------------------------------------------

------------------------------------------------------------

--------------------------DSHE-----------------STES---------

------------VE-----------DLFVAP-NQANSFM-APQM--GS-----VYVPARG

NGHSYYN--FMR-KVKSPAERRAETCEDYSPCRFYAYRHGFQQAFQRYFG-----ARNPP

Q-----------------------------------------------------------

---------------------------------RPAVTR-RY

>Dicentrarchus-labrax-mgpa

------------------------------------------------------------

-----------------MRS-LLQ-FLALCAAISL--CV----CY---------------

------------------------------------------------------------

------------------------------------------------------------

--------------------------ESHE-----------------STES---------

------------IE-----------DLFVSP-NQANSFI-TPAR--GN-----VYSPPRG

---NNYN--FMR-TVKSQAERRAESCEDYSPCRLYAYRHGYQQAYQRYFA-----SRTQP

Q-----------------------------------------------------------

---------------------------------RPAGAR-RY

>Neolamprologus-brichardi-mgpa

------------------------------------------------------------

-----------------MRS-LLR-LLALCAAVSL--CI----CY---------------

------------------------------------------------------------

------------------------------------------------------------

--------------------------DSHE-----------------STES---------

------------AE-----------DLFVPP-NRANSFI-PPQR--GN-----IYIPSRG

NGFGSYN--FRR-TFKSPAERRAEICEDYSPCRFYAYRYGAQHAYNRYFG-----ARSQP

R-----------------------------------------------------------

---------------------------------RPAVTR-RY

>Oryzias-sinensis-mgpa

------------------------------------------------------------

-----------------MRS-LLQ-CLALCAAVSF--CG----CY---------------

------------------------------------------------------------

------------------------------------------------------------

--------------------------DSHE-----------------STES---------

------------FE-----------DLFVPR-NRANSFI-TPQR--RN-----VFVPPRS

IGINQFN--VWR-PRKSPAEMQAETCEDFSPCRLYAYRFGYQQAYRRYFG-----LGGRN

RLP---------------------------------------------------------

------------------------------QSYRPAGSR-GF

>Oryzias-latipes-mgpa

------------------------------------------------------------

-----------------MRS-LLQ-CLALCAAVSF--CA----CY---------------

------------------------------------------------------------

------------------------------------------------------------

--------------------------DSHE-----------------STES---------

------------FE-----------DLFVPR-NRANSFI-TPQR--RN-----VFVPPRS

IGINQFN--VWR-PRKSPAEMQAETCEDFSPCRLYAYRFGYQQAYRRYFG-----LGGRN

RLP---------------------------------------------------------

------------------------------QSYRPAGSL-RY

>Xiphophorus-couchianus-mgpa

------------------------------------------------------------

-----------------MRS-LLQ-FLALCAVVSL--CV----CY---------------

------------------------------------------------------------

------------------------------------------------------------

--------------------------DSNE-----------------SNES---------

------------RE-----------DLFVPP-NRANSFI-QGSR--RT--------TPRA

---NLYN--QYF-SRKSPAEIRAETCEDYSPCRFYAYRFGVQHAYQRYFG-----GRNPP

QQQQQQ------------------------------------------------------

----------------------------QQQRYRPMGTR-RY

>Gadus-morhua-mgpa

------------------------------------------------------------

-----------------MEG-LLL-SVVLCTLLSL--SL----CY---------------

------------------------------------------------------------

------------------------------------------------------------

--------------------------DSQE-----------------STES---------

------------FE-----------DVFMSP-NQANSFF-NPYQ--GN-----PYQNLPR

NYYNNYN--LMR-TIKSPAERRAETCEDYSPCRFYAFRHGFQQAYQRYFG-----SRNPA

QTPVQAPVRSPVRSPVQAPIRNPIPSPVQSRGRNPVRNPVRSPVRNPVRSPVRNPVRGPV

RGPVRNPVVAPPRSQALNRGRSPVRNLVQNPVWNPFGIR-QY

>Scleropages-formosus-mgpb

------------------------------------------------------------

-----------------MKT-LLQ-CVALYVLLAL--CL----CY---------------

------------------------------------------------------------

------------------------------------------------------------

--------------------------DSHE-----------------SQES---------

------------FE-----------DLFLNG-RRASSFF-HPRR--GS-----TY-----

---NSYN--YRR-LVKSPAERRAETCEDFSPCRFYAGRYGYQLAYQRYFA-----ARRPW

D-----------------------------------------------------------

--------------------------------------R-KY

>Scleropages-formosus-mgpa

MDDRRQEGESMGGAINVEEGGEVSPHTYKSWVTNNVKSSQPEKDPEEIKKTNQARTAKPP

HFPNVPTD--------RMKA-ALQ-CVTLSVILAL--CI----CY---------------

------------------------------------------------------------

------------------------------------------------------------

--------------------------ESQE-----------------SDES---------

------------FE-----------DFFVNP-SRANSFL-HNQE--RS----------PS

SNYDNYR--YGR-VVKSQAERQTEICEDYSPCRVYAYRNGYKQAYQKYFS-----NRNSG

N-----------------------------------------------------------

--------------------------------------S-RY

>Denticeps-clupeoides-mgpa

------------------------------------------------------------

-----------------MRT-LLQCCTVLCVAITL--TV----CY---------------

------------------------------------------------------------

------------------------------------------------------------

--------------------------DSHE-----------------SNES---------

------------YE-----------DLFVSP-SRANTFI-NRPR--GH-----SYGSSSS

SRGSTYS--YRR-PVKSPVEIRSEICEDYSPCRLFAHRYGYQMAYQTYFG-----NRQPV

ANP---------------------------------------------------------

--------------------------------------R-RF

>Tetraodon-nigroviridis-mgpa

------------------------------------------------------------

-----------------MRS-PLQ-VVVFCFAICL--CV----CY---------------

------------------------------------------------------------

------------------------------------------------------------

-------------------------DDSDE-----------------SSES---------

------------LE-----------DLLLPP-NQANAFI-VPRR--GS-----VYGHTSQ

GGFRQSS--FRR-RIKSPLELHAETCEDYFPCRLYAFRHGFRQAYRRYFG-----FQNRP

H-----------------------------------------------------------

---------------------------------RPAMIH-R-

>Ictalurus-punctatus-mgpa

------------------------------------------------------------

-----------------MKS-VLR-CVTLCVILAI--AV----CF---------------

------------------------------------------------------------

------------------------------------------------------------

--------------------------ESDE-----------------SNES---------

------------LE-----------DLILNR-YRANTFM-NSPG--RN-----NYNT---

-------Y-RWG-VFKSPAERRSEICEDNFKCRLMARRYGPQFAYQKYFG-----GQRVN

NNG---------------------------------------------------------

--------------------------------------L-RY

>Astyanax-mexicanus-mgpa

MA----------------------------------------------------------

-----------------AKT-VLW-CVMACVIMAI--AA----SY---------------

------------------------------------------------------------

------------------------------------------------------------

--------------------------DSQE-----------------SHES---------

------------ME-----------DVFLNP-YRANSFM-SPSY--GQYN----------

------T--YRQ-RVKSPAELRSEICEDYYPCRTFANRYGYQLAYNTYFG-----AGQAQ

R-----------------------------------------------------------

----------------------------------GSNTR-RY

>Danio-rerio-mgpa

------------------------------------------------------------

-----------------MCV-SPQ-CVFLCVVLALGAAA----AY---------------

------------------------------------------------------------

------------------------------------------------------------

--------------------------DSQE-----------------SRES---------

------------LE------------VFVNP-YQANAFM-RNTQ--HN-----PY-----

---------IYR-RMKTPAERRAEVCEDFSPCRVFALRYGSQVAYQTFFS-----PQQLR

A-----------------------------------------------------------

----------------------------------NQQLR-RY

>Lepisosteus-oculatus-Mgp

------------------------------------------------------------

-----------------MNA-LLQ-LAVLSLLATL--CV----SY---------------

------------------------------------------------------------

------------------------------------------------------------

--------------------------DSYE-----------------SNES---------

------------LE-----------DIFIGS-RRANSYM-RPVQ--PG-----SYY----

---------PRR-ILKSPVEIRTEICEDSLQCKRYAMYHGYQQAYERYFG-----GPAQS

GRRGRG------------------------------------------------------

---------AQPR-----------------------------

>Raja-clavata-Mgp1

------------------------------------------------------------

-----------------MRT-LIL--LGLCGLAAL--CA----A----------------

------------------------------------------------------------

------------------------------------------------------------

--------------------------DSSE-----------------SNEI---------

------------DD-----------AMFLRR-RDAHFFM-RPAR--PS-----NPWE---

----------RM-RVKSPYEVNREQCEEFRPCDMLARQIGHQQAYGRFFG-----YAQPQ

SNGYRR------------------------------------------------------

---------QRSHRQ-------------RGSRSRQHYYR--Y

>Amblyraja-radiata-Mgp1

------------------------------------------------------------

-----------------MRT-LIL--LGLCGLVAL--CA----A----------------

------------------------------------------------------------

------------------------------------------------------------

--------------------------DSSE-----------------SNEI---------

------------DD-----------AMFLRR-RDANFFM-RPAR--PS-----NPWE---

----------RM-RIKSPYELNREQCEEFRPCDMLARQIGHRQAYGRYFG-----NAQPQ

ANGYRR------------------------------------------------------

---------QRSHRQ-------------RGSRSRQHYYR--Y

>Leucoraja-erinacea-Mgp1

------------------------------------------------------------

-----------------MRT-LIL--LGLCGLAAL--CA----A----------------

------------------------------------------------------------

------------------------------------------------------------

--------------------------DSSE-----------------SNEI---------

------------DD-----------AMFLRR-RDAHYFM-RPSR--PS-----NPWE---

----------RM-RAKSPYELNREQCEEFRPCDMLARQIGHRQAYGRFFG-----NAQPQ

ANGYRR------------------------------------------------------

---------QRSHRQ-------------RGSRSRQHYYR--Y

>Galeorhinus-galeus-Mgp1

------------------------------------------------------------

------------------------------------------------------------

------------------------------------------------------------

------------------------------------------------------------

--------------------------DSSE-----------------SNEI---------

------------ED-----------VLFLGR-QDANSFM-RQPR--PP-----NHWD---

---------SRD-RFKSPRERTREKCEEYRPCERLARQVGLKRAYGKYFG-----NRRQR

PSTSGR------------------------------------------------------

---------LRPRKY-------------RASRYRNHHYR--Y

>Prionace-glauca-Mgp1

------------------------------------------------------------

-----------------MRT-LIL--LSICALAAL--CG----A----------------

------------------------------------------------------------

------------------------------------------------------------

--------------------------DSSE-----------------SNEI---------

------------DD-----------VLFLGR-RDANSFM-KYPQ--LP-----NHWD---

---------SRD-RYRSPRERTRERCEEYRPCERLARQVGLKRAFGKYFG-----SRRQR

LSTSGR------------------------------------------------------

---------LRPRKH-------------RASYYRNHHYR--Y

>Scyliorhinus-canicula-Mgp1

------------------------------------------------------------

-----------------MKT-LVF--LSVCALAAV--CT----A----------------

------------------------------------------------------------

------------------------------------------------------------

--------------------------DSSE-----------------SNEI---------

------------DD-----------VLFLGR-RDAHSFM-RQPR--PP-----HHWD---

------S--SRV-RYKSPREMTREICEEHRPCERLARQVGLKRAYGRYFG-----GRRQR

PSSYER------------------------------------------------------

---------MRPRKH-------------RDTRYRNHHYR--F

>Rhincodon-typus-Mgp1

------------------------------------------------------------

----------------------------------M--AD----K----------------

------------------------------------------------------------

------------------------------------------------------------

--------------------------DSSE-----------------SNEI---------

------------DD-----------LLFLGR-RDANSFM-RQPR--LP-----NYWD---

---------SRD-RFKYPYEINREMCEEYQPCERLATQVGLKQAFGKYFA-----NGRQR

TSRYRR------------------------------------------------------

---------LKPRRY-------------RGSRSRRQHYR--Y

>Callorhinchus-milii-Mgp1

------------------------------------------------------------

-----------------MRI-LLL--LMLSVLTAI--CV----A----------------

------------------------------------------------------------

------------------------------------------------------------

--------------------------DSSE-----------------SNEI---------

------------DE-----------ALFIKR-RDANSFV-RQAK--RH-----SPWE---

------S--SRD-RFKTLRERNRERCEEYRPCDRLARQVGLKRAIGKFFR-----SGRQR

FSGYRR------------------------------------------------------

---------LRAGRN-------------RRLRKNNRNRRRRF

>Gasterosteus-aculeatus-bgp1a

------------------------------------------------------------

-----------------MKT-LAV--LLLCSLAVI--CL----TS---------------

------------------------------------------------------------

------------------------------------------------------------

----------------------DAATASQP-------------------DSP-----A--

------------QE-----------GLFVER-QQASAVA-RQ-R--RA------------

---------AGQ-LSLTQLESLTEVCEANLACEDMMDTHGIIAAYTAYYG-----PVPY-

------------------------------------------------------------

------------------------------------------

>Anabas-testudineus-bgp1a

------------------------------------------------------------

-----------------MKT-LII--LVLCSLAVV--CL----TS---------------

------------------------------------------------------------

------------------------------------------------------------

----------------------DASTGSQP-----------------AGDNP-----A--

------------QE-----------GLFVER-DEASNVV-RQ-K--RA------------

---------AAQ-LTQFQLESLREVCEANLACEHMMDTNGIIAAYTAYYG-----PIPY-

------------------------------------------------------------

------------------------------------------

>Dicentrarchus-labrax-bgp1a

------------------------------------------------------------

-----------------MKT-LAI--LVLCSLAVI--CL----TS---------------

------------------------------------------------------------

------------------------------------------------------------

----------------------DASAGSQP-----------------ASDNL-----A--

------------QE-----------GLFVER-EQASTVV-RQ-K--RA------------

---------AGD-LSLTQLESLREVCEANLACEDMMDTQGIIAAYTAYYG-----PIPY-

------------------------------------------------------------

------------------------------------------

>Diplodus-puntazzo-bgp1a

------------------------------------------------------------

-----------------MKT-LAI--LVLCSLAAI--CL----TS---------------

------------------------------------------------------------

------------------------------------------------------------

----------------------DASTGSQP-----------------AXDNP-----A--

------------DE-----------GMFVER-DQASAVV-RQ-K--RA------------

---------AGQ-LSLTQLESLREVCELNLACEHMMDTEGIIAAYTAYYG-----PIPY-

------------------------------------------------------------

------------------------------------------

>Neolamprologus-brichardi-bgp1a

------------------------------------------------------------

-----------------MKT-VAI--LALCFLVVI--CV----AS---------------

------------------------------------------------------------

------------------------------------------------------------

----------------------DAATDPQP-----------------AGDNP-----A--

------------EE-----------GLFVER-EQASTVV-RQ-K--RA------------

---------AGQ-LSL-----------------HMMDINGIIAAYTAYYG-----PIPY-

------------------------------------------------------------

------------------------------------------

>Xiphophorus-couchianus-bgp1a

------------------------------------------------------------

-----------------MKT-FIV--LVLCSLAVY--MT----SG---------------

------------------------------------------------------------

------------------------------------------------------------

----------------------TKTLNACP-----------------A------------

-------------------------GLFVEK-DEASAVV-RQ-K--RA------------

---------AAE-LSLAQLESLKEVCEANMACEHMMDTNGIIAAYTAYYG-----PIPY-

------------------------------------------------------------

------------------------------------------

>Astyanax-mexicanus-bgp1a

------------------------------------------------------------

-----------------MKAFLPL--LLLSALVAL--CV----CTGA-------------

------------------------------------------------------------

------------------------------------------------------------

----------------------DSDTKSVS-----------------VGDSP-----S--

------------EE-----------GVFVKR-DLASVVV-RQ-K--RAG-----------

---------AAD-LTQVQLESLREVCELNLACEHMMDTEGIIAAYTAYYG-----PIPF-

------------------------------------------------------------

------------------------------------------

>Danio-rerio-bgp1a

------------------------------------------------------------

-----------------MKS-LTV--LIFCCLMTV--CL----SAGL-----P--D----

------------------------------------------------------------

------------------------------------------------------------

----------------------SSDTKLLS-----------------AAESP-----N--

------------HE-----------GVFVKR-DVASIIM-RQ-K--RA------------

----GTA--PGD-LTPFQLESLREVCETNVACEHMMDTSGIITAYKTYYG-----PIPF-

------------------------------------------------------------

------------------------------------------

>Denticeps-clupeoides-bgp1a

------------------------------------------------------------

-----------------MKT-FTI--LILCAFAYV--CL----CE---------------

------------------------------------------------------------

------------------------------------------------------------

----------------------GAVDSPAT-----------------KSDSH-----G--

------------DQ-----------GVFVKK-DLASIIM-RQ-K--RA------------

-----APADPTN-LSVAQLESLREVCEADLGCEHMMDVSGIIAAYTAYYG-----PIPY-

------------------------------------------------------------

------------------------------------------

>Gadus-morhua-bgp1a

------------------------------------------------------------

-----------------MKT-IVL--LFLASLVAV--CL----SSGI-------------

------------------------------------------------------------

------------------------------------------------------------

-------------------------DEQVL-----------------QGPAH-----E--

------------EA-----------GVFVER-EEADTLV-RP-K--RA------------

---------ATE-LTLTQLESLMEVCEVDVACESMMDTAGIIAAYTAHYG-----PIPF-

------------------------------------------------------------

------------------------------------------

>Ictalurus-punctatus-bgp1a

------------------------------------------------------------

-----------------MKT-LTI--LLLSALIGL--CA----CM---------------

------------------------------------------------------------

------------------------------------------------------------

----------------------GECVCVCV------------------------------

-------------------------CVFVER-EEASIAV-RQ-K--RA------------

-----TA--AAD-LSLAQLESLREVCEANLDCENMMDTSGIIAAYTKHYG-----PIPF-

------------------------------------------------------------

------------------------------------------

>Anabas-testudineus-bgp1b

------------------------------------------------------------

-----------------MKT-LTL--LAICALLSV--CW----SMGAI---EP--DVVVD

PAADTADEAAP--ADPVDPVDPA------DPAAADSSSSSESDSTS----SSESDSNSSS

DSSDSSD--------------SDSAPSSESSESSES------------------------

--------SSSESD----SAS-SSSSSSSS-----------------SSESA-----S--

------------DE---------AAQVVVKR-DLAAALL-RK-R--RA------------

-----AP--AGT-LSPLQLESLREVCELNVACDELADTAGIVAAYTAYYG-----PVPF-

------------------------------------------------------------

------------------------------------------

>Betta-splendens-bgp1b

------------------------------------------------------------

-----------------MKT-LTL--LSICALLAA--CW----SMAAV---EP--EVVVD

PAADVTDDAAA--ADPAA-VDSS------D-SSSSSSSSSESDSAS----SSESDSNSAS

DSS-----------------------ASESSESSESA------------------SSES-

--------DSSASD----SA--ASSSSFSS-----------------SSESA-----S--

------------DE---------ASQVVVKR-DLAAALL-RS-R--RA------------

-----TP--AGD-LSPLQMESLREVCELNTACDDMADTDGIVAAYTAYYG-----PVPF-

------------------------------------------------------------

------------------------------------------

>Dicentrarchus-labrax-bgp1b

------------------------------------------------------------

-----------------MKT-LAL--LSICALLSV--CW----SMGAV---EP--EVVVD

PAADTAAEAAP--ADPAA-TDPAAPDASSSSSSSSSSSSSESDSSS-ASDSNSSSDSSAS

DSNSSSDSNSSSDSSSSSSSSSSSSSSSSSSESSESSESSESSESSESSSSESSSSSESS

SSESNSASDSSASDSTSSSSS-SSSSSSSS-----------------SSESA-----S--

------------TE---------AAQVVVKR-DLASVLL-RR-R--RA------------

-----AT--AGD-LTPLQMESLREVCELSIACDEMAETAGIVAAYTAYYG-----PVPF-

------------------------------------------------------------

------------------------------------------

>Xiphophorus-couchianus-bgp1b

------------------------------------------------------------

-----------------MKT-LTL--LSICALLSV--CW----SMGAV---EP--EVVVD

PAADTA-------ADPAA-----------DAPASSSSSSSESDSAS-----SSASDSSAS

DST------------------SDSSSSSESSESSESS------------SAE---SSESS

SSES----NSSASD----SAA-SSSSSSSS-----------------SSESA-----G--

------------AE---------ESHVVMKR-DLASVLL-RR-R--RA------------

-----A---QGN-LSPLQLESLKEVCELNVACDDMADTEGIVAAYTAYYG-----PVPF-

------------------------------------------------------------

------------------------------------------

>Oryzias-latipes-bgp1b

------------------------------------------------------------

-----------------MKT-LAL--LGLCALLSV--CR----SMSVV---EP--EVIVD

PAADAAAEVTP--APPAA-----------SSSESNSASSSESDSTSDSADSNSSSDSSAS

DST------------------SAASSESSSSESSESTSA----ASSQSSSAESSESSESS

ESDSSAASQSSESD----SAA-FASSSSSS-----------------SSESA-----S--

------------AEAAS------DSQVVMKR-DLAAVLL-RR-R--RA------------

-----AS--GGT-LTPLQMESLKEVCELNVACDEMADTEGIVAAYTAYYG-----QVPF-

------------------------------------------------------------

------------------------------------------

>Neolamprologus-brichardi-bgp1b

------------------------------------------------------------

----------------------------------------------AV---EP--EVVVD

PDADTAADVAP--ADPAA-SDSS--------SASDSSSSSESDSAS----SDSSSDSSAS

DSS-----------------------SSESSESSESSQ-----------SSESSESSESS

ES------DSSASD----SGSDSSSSSSSS-----------------SSESA-----S--

------------SEVAMFD--SSANPVVMKR-DLAAVLL-RS-R--RA------------

-----AP--AGN-LSPLQLESLREVCELNVACDEMADTSGIVAAYTAYYG-----PVPF-

------------------------------------------------------------

------------------------------------------

>Gasterosteus-aculeatus-bgp1b

------------------------------------------------------------

-----------------MKT-LTL--LSICALLSV--SC----SMGGF---EP--DVVVD

LADDTAVDSDA--ADLAD-ADVAD-------PADLDDLDDQADPDD----PDATPASPAS

DSS-S--DSSASD-------SNSSSDSTSSSESSESS------------------SSE--

--------SNSASD--------SSSSSSSS-----------------SSESV-----S--

------------EEVPTEPEPEPEPEVIMKR-DLAAVLL-RR-R--RA------------

-----AP--AGD-LSPLQLESLREVCELNDGCDEMAETAGIVAAYVAYYG-----AVPF-

------------------------------------------------------------

------------------------------------------

>Gadus-morhua-bgp1b

------------------------------------------------------------

-----------------MKT-LVL--LSICGLLTV--CW----TTGVV---EP--EAVDD

PDLDDAGEAAPDVADPYA------A------PDADTPSSSESDSPS--------------

DSSS-----------------SSSSSSSSSSESSESD------------------SAAS-

--------DSSSSS--------SSSSSSSS-----------------SSESS-----S--

------------SEATGAPALAEAPQVIMKR-DLAAVLL-RR-R--RA------------

---------TST-LSPLQTESLREVCELHDGCDEMAETEGIVAAYTAYYG-----PVPF-

------------------------------------------------------------

------------------------------------------

>Ictalurus-punctatus-bgp1b

------------------------------------------------------------

-----------------MKT-FTL--LILFSVLSA--CM----STG--------------

------------------------------------------------------------

------------------------------------------------------------

------------------------------------------------------------

--------------ATAA-----PDHVMVKR-SLATALL-RRHR--RA------------

----GTP--VAD-LTPVQLESLREVCEVNIACEHMSDTEGIVAAYTAYYG-----PIPF-

------------------------------------------------------------

------------------------------------------

>Astyanax-mexicanus-bgp1b

------------------------------------------------------------

-----------------MKT-FTL--LVLFGVLAA--CM----SMGVL----P--DVVVD

PEADAA--AAP--ADPVD------------------ADPSVSDSSS------ASDSSSAS

DSS-------------------SASDSSSASDSTSDS------------------A----

--------SDSASD--------SASDSSSS-----------------ESNSA-----S--

------------EEATPG-----PEHVVVKR-SVAASML-RRHR--RA------------

----GTP--AAA-LTPVQLESLREVCEVNMACDEMADTNGIVAAYTTYYG-----PVPF-

------------------------------------------------------------

------------------------------------------

>Denticeps-clupeoides-bgp1b

------------------------------------------------------------

-----------------MKT-FAL--LAIFGVLSV--CL----SMGV-----P--QAVED

YGLLST--AGP--PDTDG-------------SDSSSASSSASDSSA-------------S

DSTS-----------------SSESNSDSASDSASDS------------------SSD--

--------STSSSD--------STSDSASS-----------------SSESH-----S--

------------SEENP------APEVVVKR-DVAAALL-KRPR--RA------------

-----TV--AAD-LSPVQLESLREVCEANLACEDMAETAGIIAAYTAYYG-----PITL-

------------------------------------------------------------

------------------------------------------

>Danio-rerio-bgp1b

------------------------------------------------------------

-----------------MKT-LGL--LSVCALLSV--CA----SMGVYTEAEPAAEVAVD

VVVPET--GAP--VDTAS-------------SSSSSSSASDSDSAS-------SESNSAS

DSA-------------------SDSASDSASDSTDSA-----------------------

--------SDSTSD----SA--SDSSSSSS-----------------ESNSA-----S--

------------AEGTP------APHVLLKR-SVAASLL-RR-R--RA------------

----GTP--AAD-LTPVQLESLREVCEVNLACEHMAETAGIVAAYTAYYG-----KIPY-

------------------------------------------------------------

------------------------------------------

>Scleropages-formosus-bgp1b

------------------------------------------------------------

-----------------MKT-FAI--LVICGVLSV--CL----STGDL--AEP--DA---

PSAEVT-DPDPE-PDPDT--------------DADAASPTASDSTS----------DSTS

DSS-------------------SSSASDSASDSASDS------------------AS---

--------SESASD----SAS-DSASSSAS-----------------DSAST-----S--

------------AE-----------EVLVKR-DLASQLM-KRRR--RA------------

----AVA--PAD-LSPTQLESLWEVCELNLACDDMADTAGIVAAYTQYYG-----PVPF-

------------------------------------------------------------

------------------------------------------

>Scleropages-formosus-bgp1a

------------------------------------------------------------

-----------------ICA-RAI--LSFCAFACG--CL----RGFF-------------

------------------------------------------------------------

------------------------------------------------------------

----------------------FFFCACVS-----------------TYFTAWVSVT---

-------------------------EVFVKR-DLASALM-RQ-K--RAFT---A------

---KTTA--PAD-LTLTQLESLREVCEVNLACEHMAETAGIVAAYTAYYG-----PIPF-

------------------------------------------------------------

------------------------------------------

>Lepisosteus-oculatus-Bgp1

------------------------------------------------------------

-----------------------X--SVYLSVCLF--IL----CPSRL------------

----------------LL------------------------------------------

------------------------------------------------------------

----------------------DNSDVSVS-----------------APDSH-----S--

------------TE-----------EVFVKR-DLASSFV-KRLK--RNIG---AAGG--A

--AGGAG--GAG-LSLQQLESLREVCEVNLACEHMAETAGIVAAYTQYYG-----PIPY-

------------------------------------------------------------

------------------------------------------

>Erpetoichthys-calabaricus-Bgp1

------------------------------------------------------------

-----------------MRN-LTL--AVLCAVIAI--CL----CH---------------

------------------------------------------------------------

------------------------------------------------------------

----------------------GDSDNSVS-----------------APDSH-----S--

------------SE-----------DFFVKR-DTASSFV-KRLK--RN------------

----------AN-YSPQQLESLREVCEVNLACEHMAETAGILAAYQQYYG-----PIPF-

------------------------------------------------------------

------------------------------------------

>Acipenser-naccarii-Bgp1

------------------------------------------------------------

-----------------MKT-FTA--ILLLSLITL--AL----CT---------------

------------------------------------------------------------

------------------------------------------------------------

----------------------ADSDASLS-----------------APDSQ-----S--

------------AE-----------DFFVKR-DVASSFV-TRRK--KR------------

-----NA--DLT-LSPQKLESLSEVCELNTACNDLSDTVGIVAAYQKHFG-----PIPV-

------------------------------------------------------------

------------------------------------------

>Bos-taurus-Bgp1

------------------------------------------------------------

-----------------MRT-PML--LALLALATL--CL----AG---------------

------------------------------------------------------------

------------------------------------------------------------

----------------------R-ADAKPG-----------------DAESG--------

------------KG-----------AAFVSK-QEGSEVV-KRLR--RYLD---HW-----

-----LG--APA-PYPDPLEPKREVCELNPDCDELADHIGFQEAYRRFYG-----PV---

------------------------------------------------------------

------------------------------------------

>Rousettus-aegyptiacus-Bgp1

------------------------------------------------------------

-----------------MRS-PVL--LALLGLAAL--CL----AE---------------

------------------------------------------------------------

------------------------------------------------------------

----------------------Q-ADAKPP-----------------GAESS--------

------------RG-----------TAFVSK-QEGSEVV-KRLR--RYLD---HG-----

-----LG--APG-PYPDPLEPKREVCELNPDCDELADHIGFQEAYRRFYG-----PV---

------------------------------------------------------------

------------------------------------------

>Pan-troglodytes-Bgp1

------------------------------------------------------------

-----------------MRA-LTL--LALLALAAL--CI----AG---------------

------------------------------------------------------------

------------------------------------------------------------

----------------------Q-AGAKPS-----------------GAESS--------

------------KG-----------AAFVSK-QEGSEVV-KRPR--RYLY---QW-----

-----LG--APV-PYPDTLEPRREVCELNPDCDELADHIGFQEAYRRFYG-----PV---

------------------------------------------------------------

------------------------------------------

>Homo-sapiens-Bgp1

------------------------------------------------------------

-----------------MRA-LTL--LALLALAAL--CI----AG---------------

------------------------------------------------------------

------------------------------------------------------------

----------------------Q-AGAKPS-----------------GAESS--------

------------KG-----------AAFVSK-QEGSEVV-KRPR--RYLY---QW-----

-----LG--APV-PYPDPLEPRREVCELNPDCDELADHIGFQEAYRRFYG-----PV---

------------------------------------------------------------

------------------------------------------

>Oryctolagus-cuniculus-Bgp1

------------------------------------------------------------

-----------------MRA-LTL--VALLALAAL--CL----AG---------------

------------------------------------------------------------

------------------------------------------------------------

----------------------Q-AEAKPS-----------------GAESG--------

------------RG-----------SAFVSK-REGSEVV-KRAR--RQLI---DG-----

-----QG--APA-PYPDPLEPKREVCELNPDCDELADQVGLQDAYQRFYG-----PV---

------------------------------------------------------------

------------------------------------------

>Loxodonta-africana-Bgp1

------------------------------------------------------------

-----------------MRP-LTL--LALLALAAL--CL----AG---------------

------------------------------------------------------------

------------------------------------------------------------

----------------------Q-VDLKPS-----------------VADSG--------

------------KG-----------AAFVSK-RESSEVV-RRPR--RYLD---QV-----

-----LG--APA-PYPDPLELKKEVCELNPDCDELADHIGFHEAYRRFYG-----TV---

------------------------------------------------------------

------------------------------------------

>Felis-catus-Bgp1

------------------------------------------------------------

-----------------MRP-LTI--LALSALAVL--CLC--PAG---------------

------------------------------------------------------------

------------------------------------------------------------

----------------------P-ADAKPS-----------------RAESG--------

------------RG-----------AAFVSK-QEGSEVV-RRLR--RYLA---PG-----

-----LG--APA-PYPDPLEPKREICELNPDCDELADHIGFQDAYRRFYG-----TV---

------------------------------------------------------------

------------------------------------------

>Monodelphis-domestica-Bgp1

------------------------------------------------------------

-----------------MKR-VLL--LSLLTLATL--CL----CK---------------

------------------------------------------------------------

------------------------------------------------------------

------------------------QDAQAG-----------------PQDSG--------

------------KR-----------TAFASK-RDSSELV--RPK--RHLY---NW-----

-----QG--LPA-PYPDPLEQKREVCELNPDCDELADHIGFSEAYRRFYG-----TA---

------------------------------------------------------------

------------------------------------------

>Apteryx-owenii-Bgp1

------------------------------------------------------------

-----------------MRT-LAL--LTLLALVAL--GL----GR---------------

------------------------------------------------------------

------------------------------------------------------------

----------------------S------------------------GKGRP--------

------------GA-----------SAFVSR-RASAELV-RRHK--RNFA---VG-----

-----SS--Y-G-AAPDPLEAKREVCELNPDCDELADHIGFQEAYRRFYG-----PVV--

------------------------------------------------------------

------------------------------------------

>Gallus-gallus-Bgp1

------------------------------------------------------------

-----------------MKA-AAL--LLLAALLTF--SL----CR---------------

------------------------------------------------------------

------------------------------------------------------------

--------------------------SAPD-----------------GSDAR--------

------------SA-----------KAFISH-RQRAEMV-RRQK--RHYA---QD-----

-----SG--VAG-APPNPLEAQREVCELSPDCDELADQIGFQEAYRRFYG-----PV---

------------------------------------------------------------

------------------------------------------

>Rhinatrema-bivittatum-Bgp1

MLGYKFQ-----------------------------------------LETAAERREKPQ

--TSVRATRKDKA----MRS-LIL--LTLLALAMI--CL----CH---------------

------------------------------------------------------------

------------------------------------------------------------

----------------------GDADNSNS-----------------SPDSH--------

------------SK-----------EAFAAS-ASANAFI-KRLK--RQYN---PN-----

----SYG--PNN-VAQDPLEPYREVCELNPDCDELADHIGFQEAYRRFYG-----PI---

------------------------------------------------------------

------------------------------------------

>Microcaecilia-unicolor-Bgp1

------------------------------------------------------------

-----------------MRP-LTL--VTLLALAVI--CL----CH---------------

------------------------------------------------------------

------------------------------------------------------------

----------------------RDADNSNG-----------------APDAH--------

------------NT-----------EAIASNPASANAFV-KRNK--RQYN---PN-----

----SYG--ANA-VGPDPLEPYREVCELSPDCDELADQIGFEEAYRRFYG-----PL---

------------------------------------------------------------

------------------------------------------

>Xenopus-laevis-Bgp1

------------------------------------------------------------

-----------------MKL-AIL-TVLLLGAAVL--CL----GS---------------

------------------------------------------------------------

------------------------------------------------------------

----------------------KDADHSNS-----------------VGESH--------

------------SS-----------EAFISR-QESASFA-RL-K--RSYG---NN-----

-----VG--QGA-AVGSPLESQREVCELNPDCDELADHIGFQEAYRRFYG-----PV---

------------------------------------------------------------

------------------------------------------

>Xenopus-tropicalis-Bgp1

------------------------------------------------------------

-----------------MKL-AIV--LLLLGLAVL--CL----GG---------------

------------------------------------------------------------

------------------------------------------------------------

----------------------KDSQHSAS-----------------AGDSR--------

------------SS-----------EAFISR-QDSANFA-RRHK--RSYR---YN-----

-----VA--RGA-AVTSPLESQREVCELNPDCDELADHIGFQEAYRRFYG-----PV---

------------------------------------------------------------

------------------------------------------

>Anolis-carolinensis-Bgp1

------------------------------------------------------------

-----------------MKT-LIL--VALLALAAL-------------------------

------------------------------------------------------------

------------------------------------------------------------

----------------------GHTEAHSD-----------------ADNSH--------

------------DS-----------EAFVSK-RESAEVV-KRLK--QNYG---RWSQ---

---HQVA--LVP-GTRDPWEAHREVCELNPSCDELADQVGFQEAYRRFYG-----PL---

------------------------------------------------------------

------------------------------------------

>Pogona-vitticeps-Bgp1

------------------------------------------------------------

-----------------MKT-LML--VSFLAVATL-LCL---------------------

------------------------------------------------------------

------------------------------------------------------------

----------------------GGADDSAR-----------------SDDAR--------

------------SS-----------EAFLSR-RESAALV-KRDK--KDYG---RLYNI--

-----VA--APV-ATPDPLEPYREICELSPGCDELADQIGFKEAYRRYYG-----PI---

------------------------------------------------------------

------------------------------------------

>Pristis-pectinata-Mgp2

------------------------------------------------------------

------------------------------------------------------------

------------------------------------------------------------

------------------------------------------------------------

------------------------------------------------------------

------------------------------------------------------------

-----FS--VRE-IYKGPAEVNREYCDGDDSCGKGYT-----------------------

------------------------------------------------------------

------------------------------------------

>Amblyraja-radiata-Mgp2

------------------------------------------------------------

------------------------------------------------------------

------------------------------------------------------------

------------------------------------------------------------

------------------------------------------------------------

------------------------------------------------------------

-----FS--VHE-LYKGPGEVNREYCDGDENCGKGYA-----------------------

------------------------------------------------------------

------------------------------------------

>Leucoraja-erinacea-Mgp2

------------------------------------------------------------

------------------------------------------------------------

------------------------------------------------------------

------------------------------------------------------------

------------------------------------------------------------

------------------------------------------------------------

-----FS--VRE-MYKGPGEVNREYCDGDENCGKGYTHPYYSKGYGYGHGYGYGYGYAGY

YSPYYY------------------------------------------------------

---------ASS----------------------------KQ

>Scyliorhinus-canicula-Mgp2

------------------------------------------------------------

-----------------MRT-LIL--LCLCTLVAV--CMG--------------------

------------------------------------------------------------

------------------------------------------------------------

--------------------------APKE-----------FV----GPAE---------

------------KE-----------ETFMDR-QSANNFV-RRKR--HAYG---YYHHIP-

---SYES--VRE-LYKSPAEVNREYCEGDDNCGKGYP---YMAAYGKGYGYGY-GGYQGY

YAPYYQ------------------------------------------------------

---------SA-----------------------------KY

>Callorhinchus-milii-Mgp2

------------------------------------------------------------

-----------------MRT-LIV--LSLCALAVV--CLA--------------------

------------------------------------------------------------

------------------------------------------------------------

--------------------------APQE-----------------SSQA---------

------------NE-----------DTFVDK-QQANNFN-RRLK--RNVGYYPAYYQYP-

---TYES--VRE-IYKSPVEVAKEYCDGDAQCGKGYPGYMGYMGYMGYMG-----KGLGY

YRPHVK------------------------------------------------------

---------AAPAPV-------------------------KG
