# Supplementary material for: Evolution of Matrix Gla and Bone Gla Protein Genes in Jawed Vertebrates
Source: Front Genet. 2021 Mar 10;12:620659. doi: 10.3389/fgene.2021.620659 (PMC8006282; doi:10.3389/fgene.2021.620659)
Supplement: Supplementary Material 3 — Protein sequence alignment generated by HmmCleaner automatic cleaning and used to produce the phylogenetic tree reconstruction. [file Data_Sheet_3.DOCX]

%Supplementary Material 3. Protein sequence alignment generated by HMMcleaner automatic cleaning and used to produce the phylogenetic tree reconstruction (delete this line to get the fasta format file).

>Anolis-carolinensis-Mgp

--MRT-LII-LALLAVLMMA--AF--CY----ESHESIES--HE----------------

--------------YE--R-IRG-RNKSTQERQREACEDYYPCDTYALRYGYAAAYKRFF

GQRRGK---

>Pogona-vitticeps-Mgp

--MRT-LII-LALLAVLMMT--AY--CY----ESHESIES--HEFT--SPFINRRHANIF

M-R------------E--R-IRE-RSKSPQELQRERCEEYIPCERYAMRHGYVAAYKRYF

GQRRGK---

>Chrysemys-picta-belli-Mgp

--MRT-LIL-LTFLAVLMVA--AF--CY----ESHESMES--HEFV--NPFINRRNANDF

M-RAQP--RRNFIVQE--R-IRE-RNKTPQERQREICEDYNPCERYAMRHGYIAAYKRYF

GQQRGKFE-

>Alligator-sinensis-Mgp

--MRT-III-LALLAVLVMA--AF--CY----ESQESMES--HE----------------

---------------E--R-IRE-RHKSPQERHREICEDYYPCERYAYHHGYAAAYKRFF

---------

>Notechis-scutatus-Mgp

------------------------------------------------------------

---------------E--R-IRE-RRKTTQELQREECEDYSPCERYAMRHGYVAAYKRFF

GQRRGE---

>Gallus-gallus-Mgp

--MRA-LIV-LVLLAVLVMA--AT--CY----ESHESMES--HEYL--NPFLNRQRANGF

I-R------------E--R-IRE-RNKAPQERQREICEDFHLCEQYALNHGYPAAYRHYF

GRRRNK---

>Apteryx-owenii-Mgp

--MRT-LIV-LTLLAFLVMT--AT--CY----VSMYSEQS--LAFS--DPFINRRRAN--

---------------E--R-IRE-RSKAPREHQREMCEDYYPCELYAYRHGYAAAYRHYF

GRRRAK---

>Xenopus-tropicalis-Mgp

--MKT-LPV-ILLLALVAVV--AL--AY----DSYESHES--LEVY--DPFLNSRKANSF

M-NSQA--KNQ-RMNE--R-IRE-RNKSPRERQREACEDYDPCERYALRYGFSAAYKRYF

GQRRGEKK-

>Xenopus-laevis-Mgp

--MKT-LPV-ILLLALAAAV--AF--AY----DSYESHES--LEVY--DPFLNSRKANSF

M-NSQA--KNQ-RMNE--R-IRE-RNKSPRERQREACEDYDPCERYALRYGFTAAYKRYF

GQRRGEKK-

>Nanorana-parkeri-Mgp

--MKT-LGV-LLVLALAAVV--TL--AY----VRDESRES--LEV---NPFVSSRTANTF

M-SSQH--RTS-KMNE--R-IRE-RNKSPQERQREVCEDYKPCDRYALHHGYTAAYSRYF

GGRTRGK--

>Microcaecilia-unicolor-Mgp

--MRV-LFI-VALLAILAVV--TF--CS----DSHESYES--FERY--HPFVNRRKANNF

I-GLQQ--KKA-RTYE--R-IRE-LNKSPKERQREICEDHDLCELYAMRHGFQKAYKRYF

G--------

>Monodelphis-domestica-Mgp

--MKT-LLF-ITLLAALAVI--AL--CY----ESHESMES--YEM---TPFINRRKANNF

M-SPQQ--RWRAKHQE--R-VRE-RTKPTHEIHREACDDYTLCSRYAFIHGYSAAYNRYF

RQQRNI---

>Felis-catus-Mgp

--MKS-LLL-LSILAALAVA--VL--CY----ESHESMES--YEI---YPFTNRRNANTF

I-SPQQ--RWRAKAHE--R-IRE-RTKPTYEINREACDDFKLCERYAMVYGYNAAYNRYF

QQRRGGK--

>Homo-sapiens-Mgp

--MKS-LIL-LAILAALAVV--TL--CY----ESHESMES--YEL---NPFINRRNANTF

I-SPQQ--RWRAKVQE--R-IRE-RSKPVHELNREACDDYRLCERYAMVYGYNAAYNRYF

RKRRGTK--

>Mus-musculus-Mgp

--MKS-LLP-LAILAALAVA--TL--CY----ESHESMES--YEI---SPFINRRNANTF

M-SPQQ--RWRAKAQK--R-VQE-RNKPAYEINREACDDYKLCERYAMVYGYNAAYNRYF

RQRRGAKY-

>Loxodonta-africana-Mgp

--MKS-LLL-LTILAAFTVA--IL--CY----ESYESLES--HEL---SPFLNRRYANSF

I-SPQE--RWRAKAQE--R-VRE-LRKPAHELNREACEDFGICERYAMLYGYNAAYNRFF

RQRRGT---

>Ornithorhynchus-anatinus-Mgp

--MKT-LLL-LSLLVVLAAA--AV-GGY----ESHESMES--YEM---NPFIPRRNANSF

I-APRQ--RWRSRAQE--R-ARE-LSKPSHEIQREACDDYPLCQRYARNYGYRAAYTRYP

GGFRV----

>Acipenser-naccarii-Mgp

--M---------------AV--CF--SY----ESDESFDS--GE----DVFMNPYSANSF

M-NSNTNRRQTGYYYE--R-LME-RYKSPRERQRESCEEYTPCDRFARRYGYQQA---GI

GRS------

>Erpetoichthys-calabaricus-Mgp

--MQT-LLL-ATLLSLLAAI--TF--CY----DSHESIES--RE----EIFMKPYQANSF

M-NRFN-------YRQ--R-LLN-AYKRPFERQREICEDFGPCENYARFRGYNRAYQYFF

G--------

>Anolis-carolinensis-Bgp2

--------------------------------------D----E----GIKIKREVASAF

V-RRQK--RSY-PYYE--R-YYE-RFKSPMEMKQEQCENYAPCDYYSEVVGFHAAYSHFF

GHV------

>Pogona-vitticeps-Bgp2

--------------------------------------------------------ANAF

V-RRQK--RSY-PYYE--R-YYE-MFKSPMELKHEQCESYAPCDYYAEVVGFPVAYRHFF

GSA------

>Notechis-scutatus-Bgp2

--------------------------------------------------------ANTF

I-RRQK--RAY-PYSE--R-YYE-MFKSPMEMRQEQCEHYAPCNYYSEIVGFPTAYRHYF

GSI------

>Chrysemys-picta-belli-Bgp2

--MRK-LLV-LLLLTLALAA-FCY--CE---RDSKDTLESHGVE----GVKVKRETANAF

V-RRQK--RSY-PYYE--R-YYE-MYKSPMELRKEQCENYAPCDYLSDHVGFHAAYQRYF

GRF------

>Apteryx-owenii-Bgp2

--MRS-LLA-PLILALALAA-LCC--C-------------------------KREVANAF

V-RRQK--RS--DLYE--W-YFE-YYKSPMEQMHERCENYPPCDFLSDQIGFSMAYNRFF

GRY------

>Gallus-gallus-Bgp2

--MRK-LLA-PLILTLALAV-HCC--C-----------------------------ANAF

V-KRQK--RF--DMYE--W-YSE-YYKSPMEQMRERCESYPPCDYLSEQIGFPMAYNRFF

GRY------

>Rhinatrema-bivittatum-Bgp2

--MRV-LMV--LLALGLAVL------C-----------------------------ANAL

VNKRLK--RSY-DYYE--R-YYE-RFKSPLEMKKEQCENYWPCDYLSNQVGFYQAYRRYF

GPV------

>Microcaecilia-unicolor-Bgp2

--MRA-LIV--LIILGLAVL--C---------------------------------ANSL

VKKRQK--RSY-GYYE--W-IHE-QFKSPMEMKKEQCEEYWPCDYLSRQVGFHQAYRRYF

GPV------

>Xenopus-tropicalis-Bgp2

--MKE-LVI--LSLLVL--------------------------E----DVKVTRQAAHAI

I-KRVR--RGY-NYYE--R-YFP-RVKSPLELKKEQCENYSPCDQLSEWVGFYQAYQTYF

GPV------

>Xenopus-laevis-Bgp2

--MKE-LVI--LSLLVL--------------------------E----DVKVTRQAAHAV

I-KRVR--RGY-NYYE--R-YFA-RMKSPLELKKEQCENYLPCDQLSEWVGFYQAYQKYF

GPV------

>Pristis-pectinata-Bgp

-------------------------------------------------PFLEKQKANSV

M-KRPR--RSI------------------------ICESYYPCDYLANRIGFQTAYQQYF

GNY------

>Amblyraja-radiata-Bgp

------------------------------------------------------------

------------------------------------CESFSPCHHLANRVGYQNAYQQYF

G--------

>Scyliorhinus-canicula-Bgp

------------------------------------------------------------

-------------YYE--R-QHEYYYKTPYEKYKEICEAYYPCDYLANRIGYQNAYIQYF

GYY------

>Rhincodon-typus-Bgp

------------------------------------------------DPFLEKHKADSV

V-KRSR--RSIQEYYE--R-HHEYYYKTPYEKRREICESYYPCNYLANRIGFQSAYIQYF

GYY------

>Callorhinchus-milii-Bgp

--MKC-LLL--LILLGLGTL--C---------------------------------ANSM

M-KRHK--REYPNYYE--R-LREQYYKTPYERRKESCESYYPCDILANRIGYRNAYRQYF

GDY-----Y

>Anabas-testudineus-mgpa

--MRS-LLQ-FLALCAAAAL--CV--CY----DSHESTES--DE----DLFVPANRANSF

I-RPQR--R-----------------KSAAERRSETCEDFRPCRLYSFQVGRQQAYNRYF

G--------

>Gasterosteus-aculeatus-mgpa

--MRS-LLQ-LLALCAAASF--CV--CY----DSHESTES--VE----DLFVAPNQANSF

--------------------------KSPAERRAETCEDYSPCRFYAYRHGFQQAFQRYF

G--------

>Dicentrarchus-labrax-mgpa

--MRS-LLQ-FLALCAAISL--CV--CY----ESHESTES--IE----DLFVSPNQANSF

--------------------------KSQAERRAESCEDYSPCRLYAYRHGYQQAYQRYF

---------

>Neolamprologus-brichardi-mgpa

--MRS-LLR-LLALCAAVSL--CI--CY----DSHESTES--AE----DLFVPPNRANSF

--------------------------KSPAERRAEICEDYSPCRFYAYRYGAQHAYNRYF

G--------

>Oryzias-sinensis-mgpa

--MRS-LLQ-CLALCAAVSF--CG--CY----DSHESTES--FE----DLFVPRNRANSF

I-TPQR--R-----------------KSPAEMQAETCEDFSPCRLYAYRFGYQQAYRRYF

G--------

>Oryzias-latipes-mgpa

--MRS-LLQ-CLALCAAVSF--CA--CY----DSHESTES--FE----DLFVPRNRANSF

I-TPQR--R-----------------KSPAEMQAETCEDFSPCRLYAYRFGYQQAYRRYF

G--------

>Xiphophorus-couchianus-mgpa

--MRS-LLQ-FLALCAVVSL--CV--CY----DSNESNES--RE----DLFVPPNRANSF

--------------------------KSPAEIRAETCEDYSPCRFYAYRFGVQHAYQRYF

GGR------

>Gadus-morhua-mgpa

--MEG-LLL-SVVLCTLLSL--SL--CY----DSQESTES--FE----DVFMSPNQANSF

--------------------------KSPAERRAETCEDYSPCRFYAFRHGFQQAYQRYF

G--------

>Scleropages-formosus-mgpb

--MKT-LLQ-CVALYVLLAL--CL--CY----DSHESQES--FE----DLFLNGRRASSF

---------------------RR-LVKSPAERRAETCEDFSPCRFYAGRYGYQLAYQRYF

---------

>Scleropages-formosus-mgpa

------------------AL--CI--CY----ESQESDES--FE----DFFVNPSRANSF

--------------------------KSQAERQTEICEDYSPCRVYAYRNGYKQAYQKYF

---------

>Denticeps-clupeoides-mgpa

--MRT-LLQCCTVLCVAITL--TV--CY----DSHESNES--YE----DLFVSPSRANTF

I-NR-----------------RR-PVKSPVEIRSEICEDYSPCRLFAHRYGYQMAYQTYF

GNR------

>Tetraodon-nigroviridis-mgpa

--MRS-PLQ-VVVFCFAICL--CV--CY---DDSDESSES--LE----D-----------

--------------------------KSPLELHAETCEDYFPCRLYAFRHGFRQAYRRYF

G--------

>Ictalurus-punctatus-mgpa

--MKS-VLR-CVTLCVILAI--AV--CF----ESDESNES--LE----DLILNRYRANTF

M-NSPG--RN--NYNT---YRWG-VFKSPAERRSEICEDNFKCRLMARRYGPQFAYQKYF

G--------

>Astyanax-mexicanus-mgpa

MAAKT-VLW-CVMACVIMAI--AA--SY----DSQESHES--ME----DVFLNPYRANSF

M-SPSY--GQYN------T-YRQ-RVKSPAELRSEICEDYYPCRTFANRYGYQLAYNTYF

G--------

>Danio-rerio-mgpa

--MCV-SPQ-CVFLCVVLALGAAA--AY----DSQESRES--LE-----VFVNPYQANAF

M-RNTQ--HN--PY------IYR-RMKTPAERRAEVCEDFSPCRVFALRYGSQVAYQTFF

---------

>Lepisosteus-oculatus-Mgp

--MNA-LLQ-LAVLSLLATL--CV--SY----DSYESNES--LE----DIFIGSRRANSY

M-RPVQ--PG--SYY-----PRR-ILKSPVEIRTEICEDSLQCKRYAMYHGYQQAYERYF

G--------

>Raja-clavata-Mgp1

--MRT-LIL--LGLCGLAAL--CA--A-----DSSESNEI--DD----AMFLRRRDAHFF

M-RPAR--PS--NPWE-----RM-RVKSPYEVNREQCEEFRPCDMLARQIGHQQAYGRFF

G--------

>Amblyraja-radiata-Mgp1

--MRT-LIL--LGLCGLVAL--CA--A-----DSSESNEI--DD----AMFLRRRDANFF

M-RPAR--PS--NPWE-----RM-RIKSPYELNREQCEEFRPCDMLARQIGHRQAYGRYF

G--------

>Leucoraja-erinacea-Mgp1

--MRT-LIL--LGLCGLAAL--CA--A-----DSSESNEI--DD----AMFLRRRDAHYF

M-RPSR--PS--NPWE-----RM-RAKSPYELNREQCEEFRPCDMLARQIGHRQAYGRFF

G--------

>Galeorhinus-galeus-Mgp1

--------------------------------DSSESNEI--ED----VLFLGRQDANSF

M-RQPR--PP--NHWD----SRD-RFKSPRERTREKCEEYRPCERLARQVGLKRAYGKYF

GNR------

>Prionace-glauca-Mgp1

--MRT-LIL--LSICALAAL--CG--A-----DSSESNEI--DD----VLFLGRRDANSF

M-KYPQ--LP--NHWD----SRD-RYRSPRERTRERCEEYRPCERLARQVGLKRAFGKYF

G--------

>Scyliorhinus-canicula-Mgp1

--MKT-LVF--LSVCALAAV--CT--A-----DSSESNEI--DD----VLFLGRRDAHSF

M-RQPR--PP--HHWD--S-SRV-RYKSPREMTREICEEHRPCERLARQVGLKRAYGRYF

GGR------

>Rhincodon-typus-Mgp1

-------------------M--AD--K-----DSSESNEI--DD----LLFLGRRDANSF

M-RQPR--LP--NYWD----SRD-RFKYPYEINREMCEEYQPCERLATQVGLKQAFGKYF

---------

>Callorhinchus-milii-Mgp1

--MRI-LLL--LMLSVLTAI--CV--A-----DSSESNEI--DE----ALFIKRRDANSF

V-RQAK--RH--SPWE--S-SRD-RFKTLRERNRERCEEYRPCDRLARQVGLKRA-----

---------

>Gasterosteus-aculeatus-bgp1a

--MKT-LAV--LLLCSLAVI--CL-------------------E----GLFVERQQASAV

A-RQ-R--RA----------AGQ-LSLTQLESLTEVCEANLACEDMMDTHGIIAAYTAYY

GPVPY----

>Anabas-testudineus-bgp1a

--MKT-LII--LVLCSLAVV--CL-------------------E----GLFVERDEASNV

V-RQ-K--RA----------AAQ-LTQFQLESLREVCEANLACEHMMDTNGIIAAYTAYY

GPIPY----

>Dicentrarchus-labrax-bgp1a

--MKT-LAI--LVLCSLAVI--CL-------------------E----GLFVEREQASTV

V-RQ-K--RA----------AGD-LSLTQLESLREVCEANLACEDMMDTQGIIAAYTAYY

GPIPY----

>Diplodus-puntazzo-bgp1a

--MKT-LAI--LVLCSLAAI--CL------------------DE----GMFVERDQASAV

V-RQ-K--RA----------AGQ-LSLTQLESLREVCELNLACEHMMDTEGIIAAYTAYY

GPIPY----

>Neolamprologus-brichardi-bgp1a

--MKT-VAI--LALCFLVVI--C--------------------E----GLFVEREQASTV

V-RQ-K--RA----------AGQ-LSL-----------------HMMDINGIIAAYTAYY

GPIPY----

>Xiphophorus-couchianus-bgp1a

--MKT-FIV--LVLCSLA--------------------------------FVEKDEASAV

V-RQ-K--RA----------AAE-LSLAQLESLKEVCEANMACEHMMDTNGIIAAYTAYY

GPIPY----

>Astyanax-mexicanus-bgp1a

--MKAFLPL--LLLSALVAL--CV--C----------------E----GVFVKRDLASVV

V-RQ-K--RAG---------AAD-LTQVQLESLREVCELNLACEHMMDTEGIIAAYTAYY

GPIPF----

>Danio-rerio-bgp1a

--MKS-LTV--LIFCCLMTV--CL-------------------E----GVFVKRDVASII

M-RQ-K--RA------GTA-PGD-LTPFQLESLREVCETNVACEHMMDTSGIITAYKTYY

GPIPF----

>Denticeps-clupeoides-bgp1a

--MKT-FTI--LILCAFAYV--CL--C---------------------------------

-----------------------------LESLREVCEADLGCEHMMDVSGIIAAYTAYY

GPIPY----

>Gadus-morhua-bgp1a

--MKT-IVL--LFLASLVAV--CL-------------------------VFVEREEADTL

V-RP-K--RA----------ATE-LTLTQLESLMEVCEVDVACESMMDTAGIIAAYTAHY

GPIPF----

>Ictalurus-punctatus-bgp1a

--MKT-LTI--LLLSALIGL--CA--CMGECVCVCV------------CVFVEREEASIA

V-RQ-K--RA-------TA-AAD-LSLAQLESLREVCEANLDCENMMDTSGIIAAYTKHY

GPIPF----

>Anabas-testudineus-bgp1b

--MKT-LTL--LAICALLSV--C----------SSSSSESASDE--AAQVVVKRDLAAAL

L-RK-R--RA-------AP-AGT-LSPLQLESLREVCELNVACDELADTAGIVAAYTAYY

GPVPF----

>Betta-splendens-bgp1b

--MKT-LTL--LSICALLAA--C-------------SSESASDE--ASQVVVKRDLAAAL

L-RS-R--RA-------TP-AGD-LSPLQMESLREVCELNTACDDMADTDGIVAAYTAYY

GPVPF----

>Dicentrarchus-labrax-bgp1b

--MKT-LAL--LSICALLSV--C-------------------------------------

--RR-R--RA-------AT-AGD-LTPLQMESLREVCELSIACDEMAETAGIVAAYTAYY

GPVPF----

>Xiphophorus-couchianus-bgp1b

--MKT-LTL--LSICALLSV--C-------------------------------------

--RR-R--RA-------A--QGN-LSPLQLESLKEVCELNVACDDMADTEGIVAAYTAYY

GPVPF----

>Oryzias-latipes-bgp1b

--MKT-LAL--LGLCALLSV--C-------------------------------------

------------------------------ESLKEVCELNVACDEMADTEGIVAAYTAYY

GQVPF----

>Neolamprologus-brichardi-bgp1b

------------------------------------------------------------

--------RA-------AP-AGN-LSPLQLESLREVCELNVACDEMADTSGIVAAYTAYY

GPVPF----

>Gasterosteus-aculeatus-bgp1b

--MKT-LTL--LSICAL-------------------------------------------

--RR-R--RA-------AP-AGD-LSPLQLESLREVCELNDGCDEMAETAGIVAAYVAYY

GAVPF----

>Gadus-morhua-bgp1b

--MKT-LVL--LSICGLLTV--C-------------------------------------

------------------------------ESLREVCELHDGCDEMAETEGIVAAYTAYY

GPVPF----

>Ictalurus-punctatus-bgp1b

--MKT-FTL--LIL----------------------------------------------

-----------------------------LESLREVCEVNIACEHMSDTEGIVAAYTAYY

GPIPF----

>Astyanax-mexicanus-bgp1b

--MKT-FTL--LVL---------------------------------EHVVVKRSVAASM

L-RRHR--RA------GTP-AAA-LTPVQLESLREVCEVNMACDEMADTNGIVAAYTTYY

GPVPF----

>Denticeps-clupeoides-bgp1b

--MKT-FAL--L------------------------------------------------

-----------------------------LESLREVCEANLACEDMAETAGIIAAYTAYY

GPITL----

>Danio-rerio-bgp1b

--MKT-LGL--LSVCALLSV--C---------------------------------AASL

L-RR-R--RA------GTP-AAD-LTPVQLESLREVCEVNLACEHMAETAGIVAAYTAYY

GKIPY----

>Scleropages-formosus-bgp1b

--MKT-FAI--LVICGVLSV--CL------------------------------------

-----------------------------LESLWEVCELNLACDDMADTAGIVAAYTQYY

GPVPF----

>Scleropages-formosus-bgp1a

-------------------------------------------------VFVKRDLASAL

M-RQ-K--RA-------------------LESLREVCEVNLACEHMAETAGIVAAYTAYY

GPIPF----

>Lepisosteus-oculatus-Bgp1

-------------------------------------------E----EVFVKRDLASSF

V-KRLK--R--------------------LESLREVCEVNLACEHMAETAGIVAAYTQYY

GPIPY----

>Erpetoichthys-calabaricus-Bgp1

--MRN-LTL--AVLCAVIAI--CL--C----------------E----DFFVKRDTASSF

V-KRLK--RN-----------AN-YSPQQLESLREVCEVNLACEHMAETAGILAAYQQYY

GPIPF----

>Acipenser-naccarii-Bgp1

--MKT-FTA--ILLLSLITL--AL--C----------------E----DFFVKRDVASSF

V-TRRK--KR-------NA-DLT-LSPQKLESLSEVCELNTACNDLSDTVGIVAAYQKHF

GPIPV----

>Bos-taurus-Bgp1

--MRT-PML--LALLALATL--CL------------------------------------

-------------------------YPDPLEPKREVCELNPDCDELADHIGFQEAYRRFY

GPV------

>Rousettus-aegyptiacus-Bgp1

--MRS-PVL--LALLGLAAL--CL------------------------------------

-------------------------YPDPLEPKREVCELNPDCDELADHIGFQEAYRRFY

GPV------

>Pan-troglodytes-Bgp1

--MRA-LTL--LALLALAAL--C-------------------------------------

-----------------------------LEPRREVCELNPDCDELADHIGFQEAYRRFY

GPV------

>Homo-sapiens-Bgp1

--MRA-LTL--LALLALAAL--C-------------------------------------

-------------------------YPDPLEPRREVCELNPDCDELADHIGFQEAYRRFY

GPV------

>Oryctolagus-cuniculus-Bgp1

--MRA-LTL--VALLALAAL--CL------------------------------------

-------------------------YPDPLEPKREVCELNPDCDELADQVGLQDAYQRFY

GPV------

>Loxodonta-africana-Bgp1

--MRP-LTL--LALLALAAL--CL------------------------------------

-------------------------YPDPLELKKEVCELNPDCDELADHIGFHEAYRRFY

GTV------

>Felis-catus-Bgp1

--MRP-LTI--LALSALAVL--CLC-----------------------------------

-------------------------YPDPLEPKREICELNPDCDELADHIGFQDAYRRFY

GTV------

>Monodelphis-domestica-Bgp1

--MKR-VLL--LSLLTLATL--CL--C---------------------------------

-------------------------YPDPLEQKREVCELNPDCDELADHIGFSEAYRRFY

GTA------

>Apteryx-owenii-Bgp1

--MRT-LAL--LTLLALVAL----------------------------------------

----------------------------PLEAKREVCELNPDCDELADHIGFQEAYRRFY

GPVV-----

>Gallus-gallus-Bgp1

--MKA-AAL--LLLAALLTF--SL--C---------------------------------

V-RRQK--RHY-----------------PLEAQREVCELSPDCDELADQIGFQEAYRRFY

GPV------

>Rhinatrema-bivittatum-Bgp1

--MRS-LIL--LTLLALAMI--CL--C---------------------------------

----------------------------PLEPYREVCELNPDCDELADHIGFQEAYRRFY

GPI------

>Microcaecilia-unicolor-Bgp1

--MRP-LTL--VTLLALAVI--CL--C-----------------------------ANAF

V-KRNK--RQY-----------------PLEPYREVCELSPDCDELADQIGFEEAYRRFY

GPL------

>Xenopus-laevis-Bgp1

--MKL-AIL-TVLLLGAAVL--CL------------------------------------

---------------------------SPLESQREVCELNPDCDELADHIGFQEAYRRFY

GPV------

>Xenopus-tropicalis-Bgp1

--MKL-AIV--LLLLGLAVL--CL------------------------------------

---------------------------SPLESQREVCELNPDCDELADHIGFQEAYRRFY

GPV------

>Anolis-carolinensis-Bgp1

--MKT-LIL--VALLALAAL----------------------------------------

----------------------------PWEAHREVCELNPSCDELADQVGFQEAYRRFY

GPL------

>Pogona-vitticeps-Bgp1

--MKT-LML--VSFLAVATL-LCL------------------------------------

----------------------------PLEPYREICELSPGCDELADQIGFKEAYRRYY

GPI------

>Pristis-pectinata-Mgp2

------------------------------------------------------------

-----------------FS-VRE-IYKGPAEVNREYCDGDDSCGKGYT------------

---------

>Amblyraja-radiata-Mgp2

------------------------------------------------------------

-----------------FS-VHE-LYKGPGEVNREYCDGDENCGKGYA------------

---------

>Leucoraja-erinacea-Mgp2

------------------------------------------------------------

-----------------FS-VRE-MYKGPGEVNREYCDGDENC-----------------

---------

>Scyliorhinus-canicula-Mgp2

--MRT-LIL--LCLCTLVAV--C-------------------------------------

---------------------RE-LYKSPAEVNREYCEGDDNCGKGYP---YMAAY----

---------

>Callorhinchus-milii-Mgp2

--MRT-LIV--LSLCALAVV--CLA-------APQESSQA--NE----DTFVDKQQANNF

N-RRLK--R------------RE-IYKSPVEVAKEYCDGDAQC-----------------

---------
