# Supplementary material for: Evolution of Matrix Gla and Bone Gla Protein Genes in Jawed Vertebrates
Source: Front Genet. 2021 Mar 10;12:620659. doi: 10.3389/fgene.2021.620659 (PMC8006282; doi:10.3389/fgene.2021.620659)

Supplementary material 4 : Treerecs material

A. Contracted gene tree:

((((amphibian-Bgp2, sauropsid-Bgp2),(actinopterygian-Bgp1,(amphibian-Bgp1,(sauropsid-Bgp1,mammalian-Bgp1)))),chondrichtyan-Bgp),((chondrichtyan-Mgp1,chondrichtyan-Mgp2),(actinopterygian-Mgp,(amphibian-Mgp,(sauropsid-Mgp,mammalian-Mgp)))));

B. Contracted species tree:

(chondrichtyan,(actinopterygian,(amphibian,(sauropsid, mammalian))));

C. Visual output

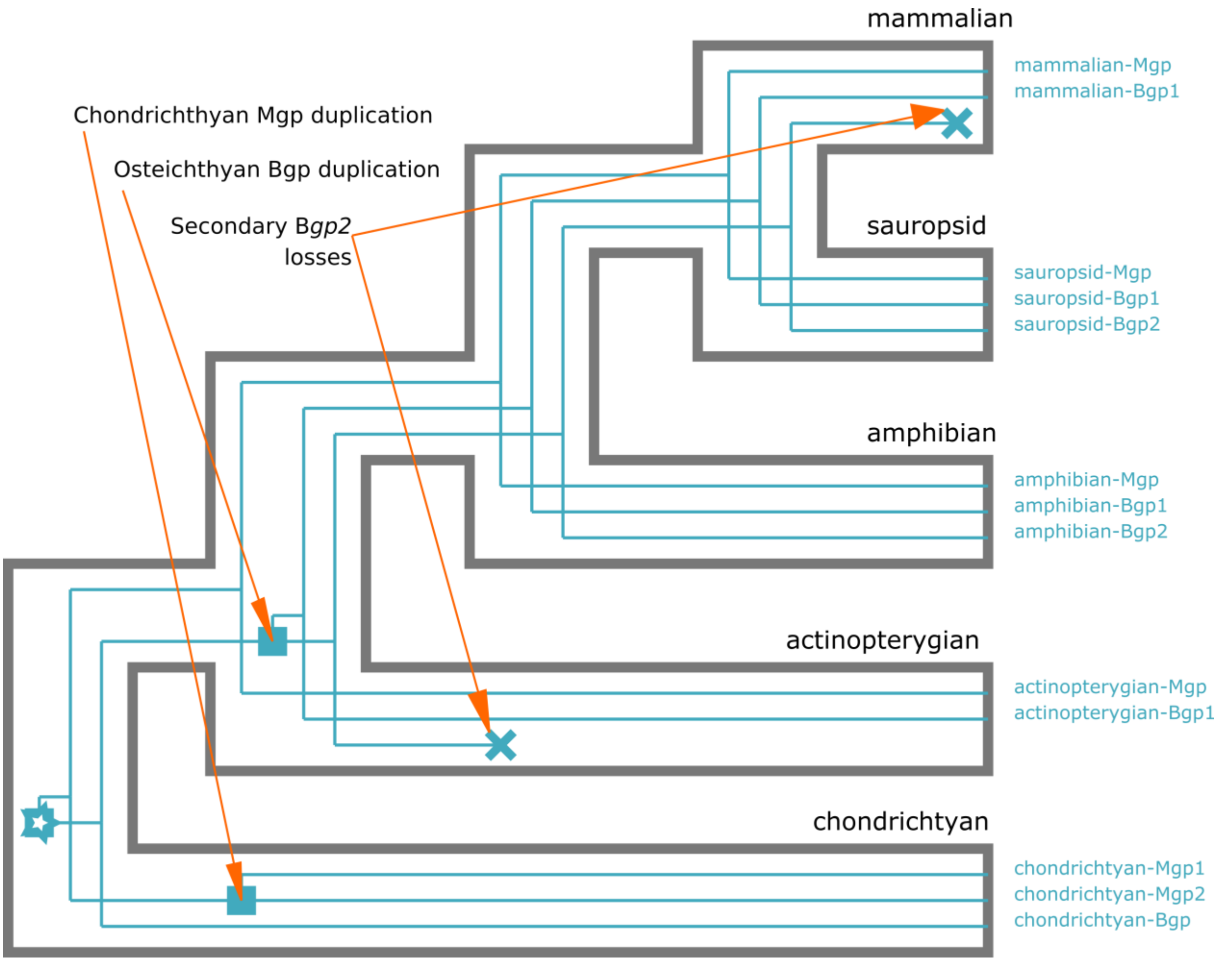

Supplement: Supplementary Material 4 — Contracted gene and species trees used with Treerecs and the visual output for the maximum parsimony scenario. [file Data_Sheet_4.PDF]
