# Supplementary figures and images for: Evolution of Matrix Gla and Bone Gla Protein Genes in Jawed Vertebrates
Source: Front Genet. 2021 Mar 10;12:620659. doi: 10.3389/fgene.2021.620659 (PMC8006282; doi:10.3389/fgene.2021.620659)

Colours and gene  
clade annotation are  
as in Figure 1

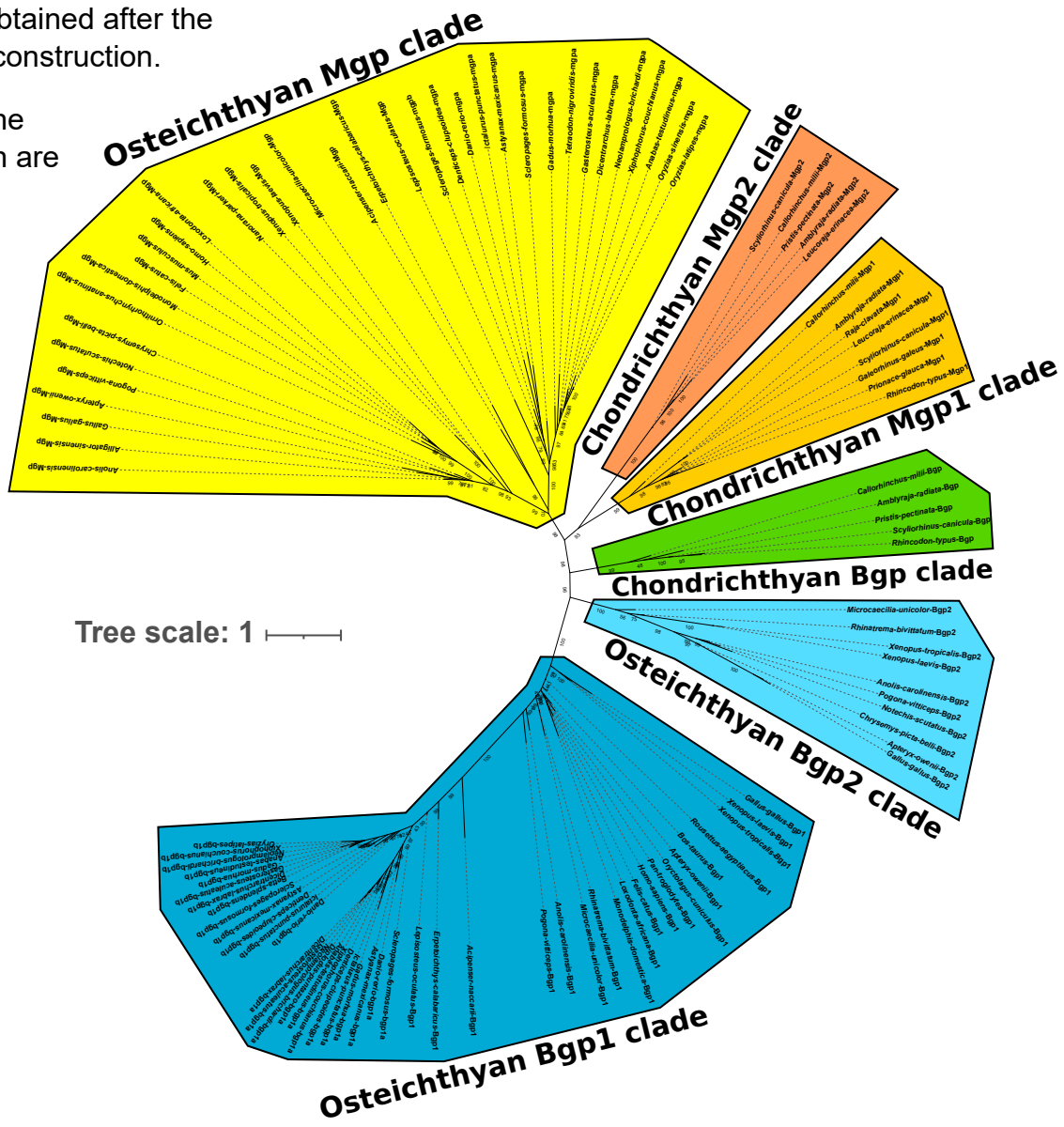

Supplement: Supplementary Material 5 — Unrooted tree obtained after the phylogenetic reconstruction. Colors and gene clade annotations are as in Figure 1. [file Data_Sheet_5.PDF]
