# Supplementary material for: Evolution of Matrix Gla and Bone Gla Protein Genes in Jawed Vertebrates
Source: Front Genet. 2021 Mar 10;12:620659. doi: 10.3389/fgene.2021.620659 (PMC8006282; doi:10.3389/fgene.2021.620659)
Supplement: Supplementary Material 6 — Alignment identifying conserved protein regions of Bgp proteins between the human Homo sapiens, the chicken Gallus gallus, the zebrafish Danio rerio, the small spotted catshark Scyliorhinus canicula, and the elephant shark Callorhinchus milii: signal peptide in the first 22 amino-acids, furin cleavage site in positions 162–166 of the alignment, core Gla domain starting on position 186. [file Data_Sheet_6.DOCX]

%Supplementary Material 6. Alignment identifying conserved protein regions of Bgp proteins between the human *Homo sapiens*, the chicken *Gallus gallus*, the zebrafish *Danio rerio*, the small spotted catshark *Scyliorhinus canicula*, and the elephant shark *Callorhinchus milii*: signal peptide in the first 22 amino-acids, furin cleavage site in positions 162-166 of the alignment, core Gla domain starting on position 186. (delete this line to get the fasta format file).

>Callorhinchus-milii-Bgp

MKCLL--LLILLGLGTLCTSRGV-------------------------------------

-------------------------------DSVENNSDVADSTENE------THSDDSA

SAQVTRNVKNGP------------------FLEKTKANSMMKRHKREYPNYYERLRE-QY

YKTPYERRKESCESYYPCDILANRIGYRNAYRQYFGDY-Y

>Scyliorhinus-canicula-Bgp

MRHLL--LITLLALVGQSCCRVISSRSDESLGDDKREAADIKGFSSHSDESLGDDKQQAD

GIEGFSSHSDESLGDDKQGSVAIEGISGRSDESLTEDSDEVANRKSKVI---GSREDDDS

VENESEADTKGP------------------FLGKREASSVVKKSKRSIQEYYERQHE-YY

YKTPYEKYKEICEAYYPCDYLANRIGYQNAYIQYFGYY--

>Homo-sapiens-Bgp1

MRALT--LLALLALAALCIAG---------------------------------------

--------------------------------------------------------QAGA

KPSGAESSKGAA------------------FVSKQEGSEVVKRPRRY---LYQWLGAPVP

YPDPLEPRREVCELNPDCDELADHIGFQEAYRRFYGPV--

>Gallus-gallus-Bgp1

MKAAA--LLLLAALLTFSLCR---------------------------------------

-----------------------------------------------------------S

APDGSDARSAKA------------------FISHRQRAEMVRRQKRH---YAQDSGVAGA

PPNPLEAQREVCELSPDCDELADQIGFQEAYRRFYGPV--

>Gallus-gallus-Bgp2

MRKLLAPLILTLALAVHCCCE---------------------------------------

--------------------------------------------------------KDPK

EPSGSPSA-ASI------------------TVEKEVANAFVKRQKRF--DMYEWYSE--Y

YKSPMEQMRERCESYPPCDYLSEQIGFPMAYNRFFGRY--

>Danio-rerio-Bgp1a

MKSLT--VLIFCCLMTVCLSAGL-------------------------------------

-----------------------------------------------------PDSSDTK

LLSAAESPNHEG------------------VFVKRDVASIIMRQKRA------GTAPGDL

TPFQLESLREVCETNVACEHMMDTSGIITAYKTYYGPIPF

>Danio-rerio-Bgp1b

MKTLG--LLSVCALLSVCASMGVYTEAE--------PAAEVA-----VDVVVPETGAPVD

TASSSSSSSSAS-----------------DSDSASSESNSASDSASDSASDSASDSTDSA

SDSTSDSASDSSSSSSESNSASAEGTPAPHVLLKRSVAASLLRRRRA------GTPAADL

TPVQLESLREVCEVNLACEHMAETAGIVAAYTAYYGKIPY
