# Supplementary material for: Evolution of Matrix Gla and Bone Gla Protein Genes in Jawed Vertebrates
Source: Front Genet. 2021 Mar 10;12:620659. doi: 10.3389/fgene.2021.620659 (PMC8006282; doi:10.3389/fgene.2021.620659)
Supplement: Supplementary Material 7 — Alignment identifying conserved protein regions of Mgp proteins between the human Homo sapiens, the zebrafish Danio rerio, and both Mgp1 and Mgp2 sequences found in chondrichthyans. Signal peptide is found in the first 20 amino-acids, conserved phosphorylation domain in the Mgp1 sequences in positions 28–36, a partially conserved ANxF domain located in position 48–51 with a F in position 42 (considered as a carboxylase docking site), core Gla domain starting on position 83 with poor conservation of the Mgp2 sequence after position 90. Note the furin cleavage site in positions 53–56 of the alignment, found in Callorhinchus milii Mgp2 sequence only. [file Data_Sheet_7.DOCX]

%Supplementary Material 7 Alignment identifying conserved protein regions of Mgp proteins between the human *Homo sapiens*, the zebrafish *Danio rerio*, and both Mgp1 and Mgp2 sequences found in chondrichthyans. Signal peptide is found in the first 20 amino-acids, conserved phosphorylation domain in the Mgp1 sequences in positions 28-36, a partially conserved ANxF domain located in position 48-51 with a F in position 42 (considered as a carboxylase docking site), core Gla domain starting on position 83 with poor conservation of the Mgp2 sequence after position 90. Note the furin cleavage site in positions 53-56 of the alignment, found in *Callorhinchus millii* Mgp2 sequence only (delete this line to get the fasta format file)

>Raja-clavata-Mgp1

M----RTLILLGLCGLAALC-----AADSSE--SNEIDDAMFLRRRDAHFFMRPAR----

------PSNPWE--RMRVKSPYEVNREQCEEFRPCDM------LARQIGHQQAYGR----

--FFGYAQPQSNGYRRQRSHRQRGSRSRQHYYR--Y

>Amblyraja-radiata-Mgp1

M----RTLILLGLCGLVALC-----AADSSE--SNEIDDAMFLRRRDANFFMRPAR----

------PSNPWE--RMRIKSPYELNREQCEEFRPCDM------LARQIGHRQAYGR----

--YFGNAQPQANGYRRQRSHRQRGSRSRQHYYR--Y

>Leucoraja-erinacea-Mgp1

M----RTLILLGLCGLAALC-----AADSSE--SNEIDDAMFLRRRDAHYFMRPSR----

------PSNPWE--RMRAKSPYELNREQCEEFRPCDM------LARQIGHRQAYGR----

--FFGNAQPQANGYRRQRSHRQRGSRSRQHYYR--Y

>Prionace-glauca-Mgp1

M----RTLILLSICALAALC-----GADSSE--SNEIDDVLFLGRRDANSFMKYPQ----

------LPNHWD-SRDRYRSPRERTRERCEEYRPCER------LARQVGLKRAFGK----

--YFGSRRQRLSTSGRLRPRKHRASYYRNHHYR--Y

>Scyliorhinus-canicula-Mgp1

M----KTLVFLSVCALAAVC-----TADSSE--SNEIDDVLFLGRRDAHSFMRQPR----

------PPHHWDSSRVRYKSPREMTREICEEHRPCER------LARQVGLKRAYGR----

--YFGGRRQRPSSYERMRPRKHRDTRYRNHHYR--F

>Callorhinchus-milii-Mgp1

M----RILLLLMLSVLTAIC-----VADSSE--SNEIDEALFIKRRDANSFVRQAK----

------RHSPWESSRDRFKTLRERNRERCEEYRPCDR------LARQVGLKRAIGK----

--FFRSGRQRFSGYRRLRAGRNRRLRKNNRNRRRRF

>Homo-sapiens-Mgp

M----KSLILLAILAALAVVTLCYESHESME--SYELNP--FINRRNANTFISPQQR---

-----WRAKVQERIRERSKPVHELNREACDDYRLCER------YAMVYGYNAAYNR----

--YFRKRRGT----------------------K---

>Danio-rerio-Mgp

MCVSPQCVFLCVVLALGAAA-----AYDSQE--SRESLE-VFVNPYQANAFMRNTQH---

--------NPY--IYRRMKTPAERRAEVCEDFSPCRV------FALRYGSQVAYQT----

--FFSPQQLRANQQLR----------------R--Y

>Callorhinchus-milii-Mgp2

M----RTLIVLSLCALAVVC-----LAAPQE--SSQANEDTFVDKQQANNFNRRLKRNVG

YYPAYYQYPTYESVREIYKSPVEVAKEYCDGDAQCGKGY--PGYMGYMGYMGYMGKG---

LGYYRPHVKAAPAPVK-------------------G

>Scyliorhinus-canicula-Mgp2

M----RTLILLCLCTLVAVC-----MGAPKEFVGPAEKEETFMDRQSANNFVRRKRHAYG

Y---YHHIPSYESVRELYKSPAEVNREYCEGDDNCGKGY--P-YMAAYGKGYGYGYG-GY

QGYYAPYYQSAK-----------------------Y

>Leucoraja-erinacea-Mgp2

F-----------------------------------------------------------

------------SVREMYKGPGEVNREYCDGDENCGKGYTHPYYSKGYGYGHGYGYGYGY

AGYYSPYYYASS--------------------K--Q
